# The Global Landscape of Genetic Variation in Parkinson’s disease: Multi-Ancestry Insights into Established Disease Genes and their Translational Relevance

**DOI:** 10.1101/2025.07.08.25330815

**Published:** 2025-07-11

**Authors:** Lara M. Lange, Zih-Hua Fang, Mary B. Makarious, Nicole Kuznetsov, Kajsa Atterling Brolin, Shannon Ballard, Soraya Bardien, Maria Leila Doquenia, Peter Heutink, Henry Houlden, Hirotaka Iwaki, Simona Jasaityte, Lietsel Jones, Johanna Junker, Rauan Kaiyrzhanov, Mathew J. Koretsky, Kishore R. Kumar, the Latin American Research Consortium on the Genetics of Parkinson's Disease (LARGE-PD), Hampton L. Leonard, Kristin S. Levine, Shen-Yang Lim, Niccoló E. Mencacci, Wael M. Y. Mohamed, Mike A. Nalls, Alastair J. Noyce, Rajeev Ojha, Njideka U. Okubadejo, Shoaib ur Rehman, Laurel Screven, Chingiz Shashkin, Sophia Sopromadze, Eleanor J. Stafford, Ai Huey Tan, Manuela Tan, Zaruhi Tavadyan, Joanne Trinh, Bayasgalan Tserensodnom, Enza Maria Valente, Dan Vitale, Nazira Zharkinbekova, Katja Lohmann, Sara Bandres-Ciga, Cornelis Blauwendraat, Andrew Singleton, Huw R. Morris, Christine Klein, the Global Parkinson’s Genetics Program (GP2)

**Affiliations:** 1 Laboratory of Neurogenetics, National Institute on Aging, Bethesda, Maryland, USA; 2 Institute of Neurogenetics, University of Luebeck, Luebeck, Germany; 3 German Center for Neurodegenerative Diseases (DZNE), Tu bingen, Germany; 4 DataTecnica, Washington, DC, USA; 5 Center for Alzheimer's and Related Dementias (CARD), National Institute on Aging and National Institute of Neurological Disorders and Stroke, National Institutes of Health, Bethesda, MD, USA; 6 Translational Neurogenetics Unit, Department of Experimental Medical Science, Lund University, Lund, Sweden; 7 Centre for Preventive Neurology, Wolfson Institute of Population Health, Queen Mary University of London, London, United Kingdom; 8 Division of Molecular Biology and Human Genetics, Faculty of Medicine and Health Sciences, Stellenbosch University; South African Medical Research Council/Stellenbosch University Genomics of Brain Disorders Research Unit, Cape Town, South Africa; 9 Department of Neuroscience and Brain Health, Metropolitan Medical Center, Manila, Philippines; 10 Department of Clinical Neurosciences, Mary Mediatrix Medical Center, Lipa, Batangas, Philippines; 11 Department of Anatomy, University of the East Ramon Magsaysay Memorial Medical Center, Quezon City, Philippines; 12 The Global Parkinson’s Genetics Program (GP2); 13 University College London, Institute of Neurology, Department of Neuromuscular Diseases, Queen Square, WC1N 3BG London, UK; 14 Department of Clinical and Movement Neurosciences, UCL Queen Square Institute of Neurology, London, UK; 15 University Hospital Schleswig Holstein, Campus Luebeck, Luebeck, Germany; 16 Center for Movement Disorders and Neuromodulation, University Hospital Duesseldorf, Duesseldorf, Germany; 17 Department of Neurology, South Kazakhstan Medical Academy, 160019, Shymkent, Kazakhstan; 18 Neurology Department and Molecular Medicine Laboratory, Concord Repatriation General Hospital and University of Sydney, Concord, NSW, 2139; 19 Translational Neurogenomics Group, Genomics and Inherited Disease Program, The Garvan Institute of Medical Research, Darlinghurst, NSW, 2010, Australia.; 20 Faculty of Medicine and Health, University of New South Wales, Sydney, NSW, Australia; 21 Division of Neurology, Department of Medicine, Faculty of Medicine, University of Malaya, Kuala Lumpur, Malaysia; 22 Department of Neurology, Northwestern University Feinberg School of Medicine, Chicago, IL, USA; 23 Department of Basic Medical Sciences, Kulliyyah of Medicine, International Islamic University Malaysia (IIUM), Kuantan, Pahang, Malaysia; 24 Clinical Pharmacology Department, Menoufia Medical School, Menoufia University, Shebin El-Kom, Menoufia, Egypt; 25 Department of Neurology, Tribhuvan University Teaching Hospital, Kathmandu, Nepal; 26 Neurology Unit, Department of Medicine, College of Medicine, University of Lagos, Lagos State, Nigeria; 27 Department of Biotechnology, University of science and Technology Bannu, Pakistan; 28 International Research Institute of Postgraduate Education, Department of Neurosurgery and Neurology, Almaty, Kazakhstan; 29 Shashkin Clinic, Almaty, Kazakhstan; 30 Department of Neurology, Ivane Javakhishvili Tbilisi State University, Tbilisi, Georgia; 31 East European University (EEU), Tbilisi, Georgia; 32 Department of Neurology, Oslo University Hospital, Oslo, Norway; 33 National Institute of Health, Yerevan, Armenia; 34 Department of Neurology, School of Medicine, Mongolian National University of Medical Sciences, Ulaanbaatar, Mongolia; 35 Department of Molecular Medicine, University of Pavia, Pavia, Italy; 36 Neurogenetics Research Center, IRCCS Mondino Foundation, Pavia, Italy; 37 UCL Movement Disorders Centre, University College London, London, UK

**Keywords:** PD, Multi-ancestry, Genetics, *LRRK2*, *GBA1*, *SNCA*, *PRKN*, causal variants, risk variants, precision medicine

## Abstract

**Background:**

The genetic architecture of Parkinson's disease (PD) varies considerably across ancestries, yet most genetic studies have focused on individuals of European descent, limiting our insights into the genetic architecture of PD at a global scale.

**Methods:**

We conducted a large-scale, multi-ancestry investigation of causal and risk variants in PD-related genes. Using genetic datasets from the Global Parkinson's Genetics Program, we analyzed sequencing and genotyping data from 69,881 individuals, including 41,139 affected and 28,742 unaffected, from eleven different ancestries, including ~30% of individuals from non-European ancestries.

**Findings:**

Our findings revealed shared and ancestry-specific patterns in the prevalence and spectrum of PD-associated variants. Overall, ~2% of affected individuals carried a causative variant, with substantial variations across ancestries ranging from <0·5% in African, African-admixed, and Central Asian to >10% in Middle Eastern and Ashkenazi Jewish ancestries. Including disease-associated *GBA1* and *LRRK2* risk variants raised the yield to ~12.5%, largely driven by *GBA1*, except in East Asians, where *LRRK2* risk variants dominated. *GBA1* variants were most frequent globally, albeit with substantial differences in frequencies and variant spectra. While *GBA1* variants were identified across all ancestries, frequencies ranged from 3·4% in Middle Eastern to 51·7% in African ancestry. Similarly, *LRRK2* variants showed ancestry-specific enrichment, with G2019S most frequently seen in Middle Eastern and Ashkenazi Jewish, and risk variants predominating in East Asians. However, clinical trials targeting proteins encoded by these genes are primarily based in Europe and North America.

**Interpretation:**

This large-scale, multi-ancestry assessment offers crucial insights into the population-specific genetic architecture of PD. It underscores the critical need for increased diversity in PD genetic research to improve diagnostic accuracy, enhance our understanding of disease mechanisms across populations, and ensure the equitable development and application of emerging precision therapies.

## Introduction

Genetic discoveries have transformed our understanding of Parkinson’s disease (PD). Genetic factors substantially contribute to PD risk and progression,^1^ comprising a spectrum ranging from rare variants with large effect sizes to common variants with small effect sizes. More than 130 independent risk loci have been identified by genome-wide association studies,^2^ and over 15 genes have been linked to monogenic PD and parkinsonism.^1,3^

A critical gap in PD genetics research is the lack of ancestral diversity.^4^ Although genetic forms of PD are found globally, approximately 75% of all genetic PD studies to date focused on European-ancestry individuals.^5,6^ Research in diverse populations is critical to uncovering unique genetic contributions to disease, which is particularly relevant for clinical trials driven by genetic discoveries, e.g., targeting glucocerebrosidase or LRRK2 kinase activity (encoded by the PD-linked genes *GBA1* and *LRRK2*, respectively). Lack of diversity may limit the global applicability of emerging opportunities, as it is unclear whether current genetic targets are relevant across ancestries or whether population-specific variants exist. Expanding genetic studies to all ancestries is essential to improve diagnostic accuracy and therapeutic development.

The Global Parkinson’s Genetics Program (GP2; https://gp2.org/)^7^ is a large-scale initiative actively addressing this gap by investigating the genetic underpinnings of PD and parkinsonism across global populations.^5,8^ The aim of this study was to investigate pathogenic and high-risk variants linked to PD and parkinsonism at a global scale using data from GP2.

## Materials and Methods

### Study design and participants

**Figure 1.**
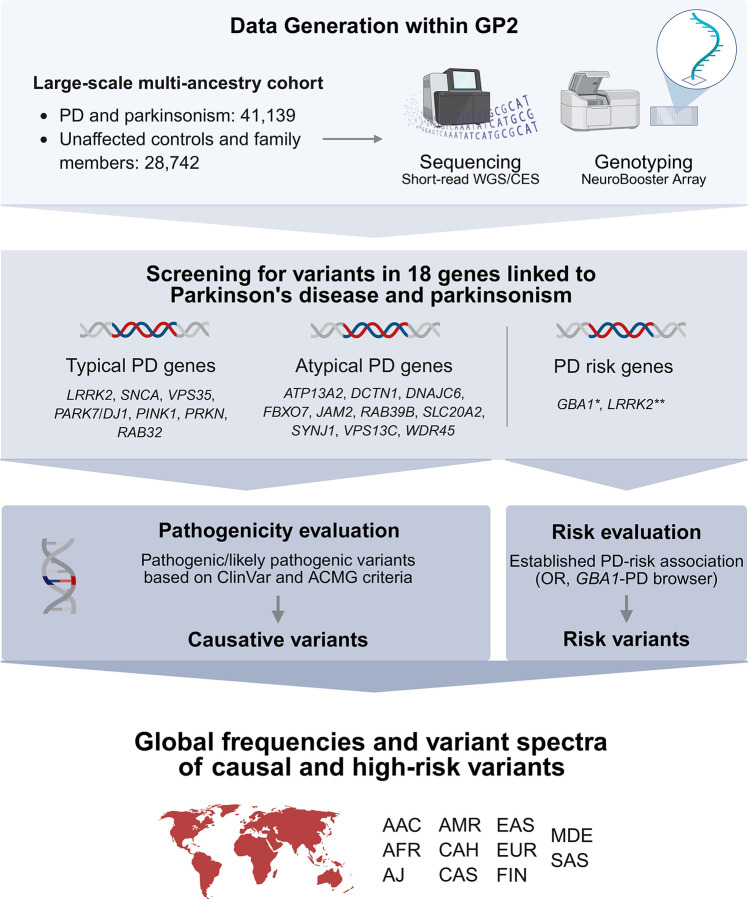
Study workflow. We used short-read sequencing and genome-wide genotyping data and investigated variants in 18 genes linked to PD and parkinsonism. Pathogenic/likely pathogenic variants according to ClinVar and/or ACMG criteria are referred to as causative variants, and selected variants with an established PD-risk association are referred to as risk variants. Figure created with BioRender. * For the purpose of this study and in the context of PD, all variants in *GBA1* (including variants causal for Gaucher’s disease) are considered PD risk variants (regardless of their Gaucher’s severity). ** Select *LRRK2* variants with established PD-risk associations included rs33949390 (chr12:40320043:G:C, R1628P) and rs34778348 (chr12:40363526:G:A, G2385R). AAC = African Admixed, ACMG = American College of Medical Genetics and Genomics, AFR = African, AJ = Ashkenazi Jewish, AMR = Latinos and Indigenous people of the Americas, CAH = Complex Admixture, CES = clinical exome sequencing, CAS = Central Asian, EAS = East Asian, EUR = European, FIN = Finnish, GP2 = Global Parkinson’s Genetics Program, MDE = Middle Eastern, OR = Odds ratio, PD = Parkinson’s disease, SAS = South Asian, WGS = whole-genome sequencing

Our study workflow is displayed in Figure 1. We used short-read sequencing data from GP2 Release 8 (DOI 10.5281/zenodo.13755496), including clinical exome and genome sequencing (WGS) data, and genome-wide Illumina NeuroBooster Array genotyping data from GP2 Release 9 (DOI 10.5281/zenodo.14510099). Data processing and quality control are described elsewhere.^9^

The sample characteristics are summarized in Table 1. The total sample comprised 69,881 individuals, including 41,139 clinically affected individuals (PD and other neurodegenerative phenotypes) and 28,742 unaffected individuals (controls, population cohorts, and unaffected family members). Both groups included genetically enriched samples, i.e., individuals specifically recruited based on their genetic status (e.g., *GBA1, LRRK2,* or *SNCA* variant carriers). Supplementary Table 1 summarizes the number of samples categorized by available genetic data, and divided by genetically determined^9^ ancestry, including African-Admixed (AAC), African (AFR), Ashkenazi Jewish (AJ), Latinos and Indigenous People of the Americas (AMR), Complex Admixture (CAH), Central Asian (CAS), East Asian (EAS), European (EUR), Finnish (FIN), Middle Eastern (MDE), and South Asian (SAS).

### Genes and variants of interest

We focused on variants in genes linked to PD and parkinsonism following the recommendations of the *MDS Task Force on the Nomenclature of Genetic Movement Disorders^3^*, including variants in i) *LRRK2, SNCA,* and *VPS35* linked to autosomal-dominant PD, ii) *DJ-1/PARK7, PINK1,* and *PRKN* linked to autosomal-recessive PD, and iii) *ATP13A2*, *DCTN1, DNAJC6, FBXO7, JAM2, RAB39B, SLC20A2, SYNJ1, VPS13C,* and *WDR45* linked to atypical parkinsonism. We included only PARK-designated genes; variants in genes linked to combined phenotypes including PD or parkinsonism (e.g., dystonia-parkinsonism) were beyond the scope of this study. We added *RAB32* S71R, identified as causal for PD after the Task Force gene list was published. We included pathogenic/likely pathogenic (according to ClinVar; https://www.ncbi.nlm.nih.gov/clinvar/) variants in these genes and also investigated PD risk-associated variants in *GBA1* and *LRRK2*, given their established translational significance. For details on pathogenicity evaluation, see Figure 1 and Supplementary Material. Copy number variation (CNV) analyses for *SNCA* and *PRKN* were done as described before.^10^ Carriers of two heterozygous pathogenic variants in recessive genes and an age at onset (AAO) ≤50 years were considered likely compound heterozygous without further validation, based on findings from previous studies.^11^

## Results

### Summary

Across all ancestries, a total of 2·1% (826/40,288) of affected individuals carried a causative and 10·8% (4,331/40,288) a risk variant (Table 2). In comparison, causative variants were observed in 0.2% (67/28,014) and risk variants in 7·5% (2,103/28,014) of unaffected individuals (Table 3).

**Figure 2.**
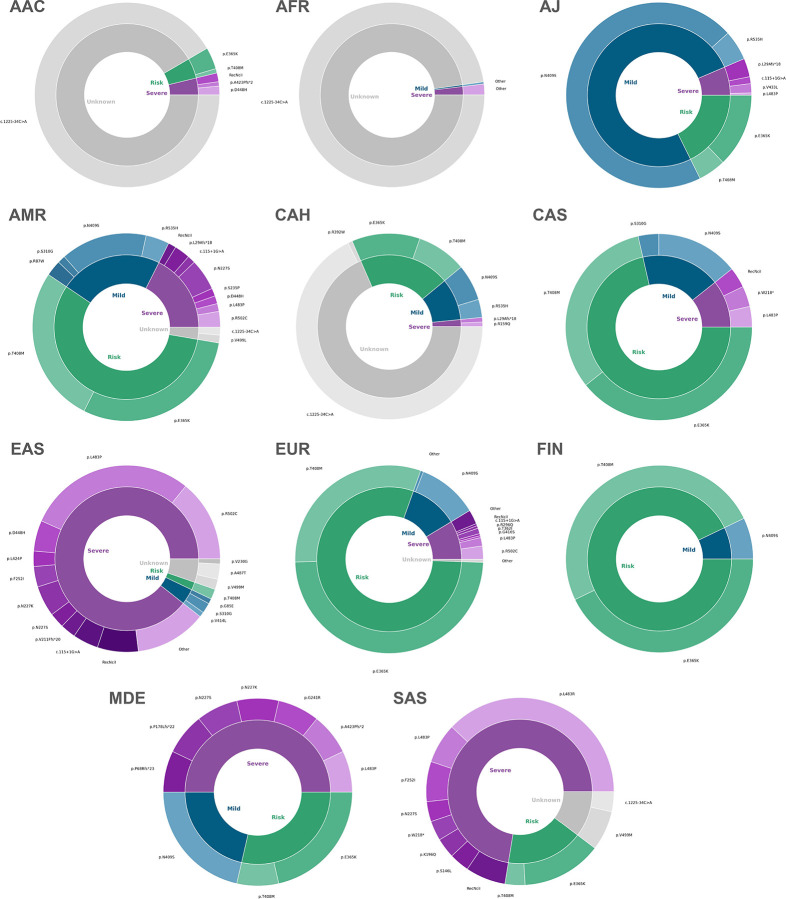
Mutational and severity spectrum of *GBA1* variants across ancestries. Donut charts illustrate the spectrum of *GBA1* variants identified across ancestries, stratified by variant severity. Severity was assessed using the *GBA1*-PD browser (https://pdgenetics.shinyapps.io/GBA1Browser/); notably, the newly identified c.1225–34C>A variant (rs3115534-G) is not yet included and was therefore grouped as unknown. Each segment size reflects the percentage of variant carriers within each ancestry. The inner colors represent variant severity categories: Severe (purple), Mild (blue), Risk (green), and Unknown (gray). The outer segments show individual variants, with color shades corresponding to the inner severity group. Due to the high number of different variants, for EAS, all severe variants with <3 carriers were grouped as “Other”, and for EUR, all variants with <10 carriers were grouped as “Other”. Similarly, due to the very small percentage of *GBA1* variants other than c.1225–34C>A in AFR, all severe and mild variants were each grouped as “Other”. AAC = African Admixed, AFR = African, AJ = Ashkenazi Jewish, AMR = Latinos and Indigenous people of the Americas, CAH = Complex Admixture, CAS = Central Asian, EAS = East Asian, EUR = European, FIN = Finnish, MDE = Middle Eastern, SAS = South Asian.

**Figure 3.**
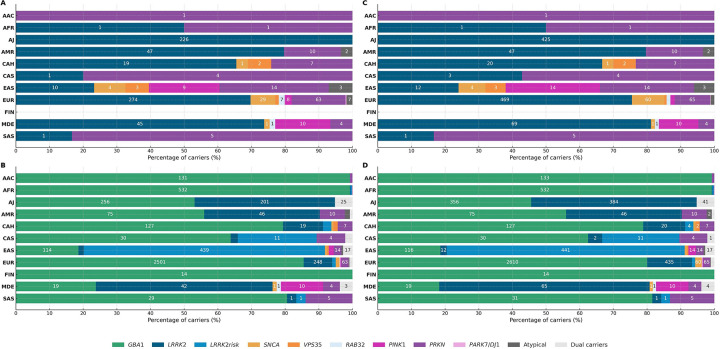
Distribution of variants in genes linked to Parkinson’s disease (PD) and parkinsonism across ancestries. Horizontal stacked bar charts displaying the percentage of identified variant carriers per gene amongst all carriers per ancestry: (A) only variants considered causal (excluding genetically enriched cohorts), (B) causal and risk variants excluding genetically enriched cohorts, and (C) causal and risk variants including genetically enriched cohorts. Numbers in bars indicate the absolute number of carriers. Dual carriers harbor variants in two different genes (e.g., *LRRK2* and *GBA1*). Atypical includes carriers of variants in *ATP13A2, RAB39B, SLC20A2,* and *WDR45*. *LRRK2* risk variants are R1628P and G2385R. AAC = African Admixed, AFR = African, AJ = Ashkenazi Jewish, AMR = Latinos and Indigenous people of the Americas, CAH = Complex Admixture, CAS = Central Asian, EAS = East Asian, EUR = European, FIN = Finnish, MDE = Middle Eastern, SAS = South Asian.

**Figure 4.**
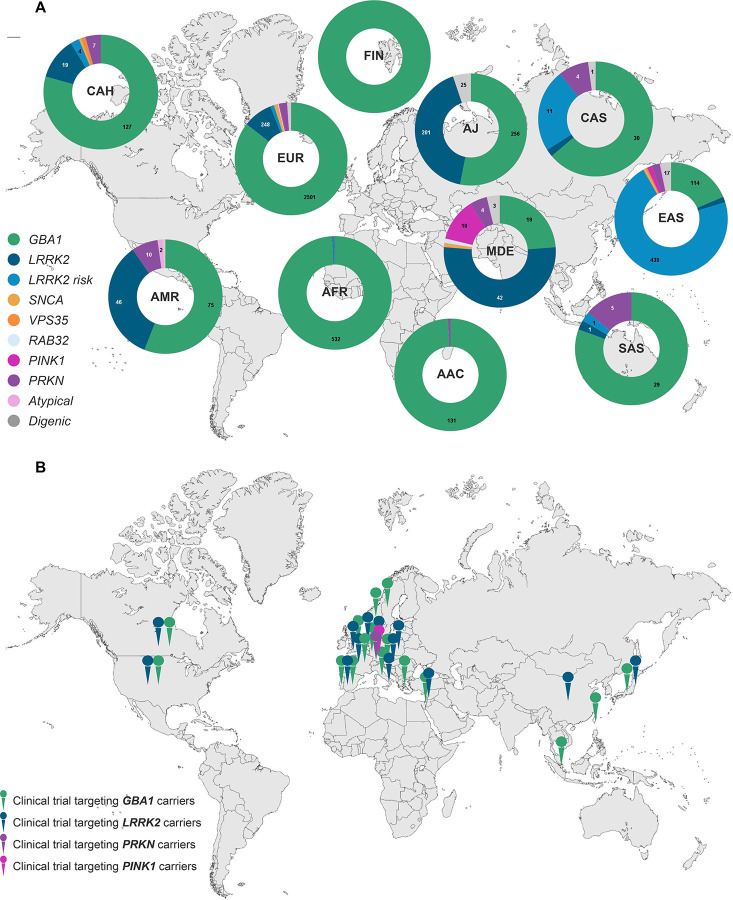
Global genetic spectrum of PD and locations of clinical trials targeting gene variant carriers. (A) Donut charts displaying the percentage of identified carriers per gene amongst all carriers per ancestry. Dual carriers harbor variants in two different genes (e.g., *LRRK2* and *GBA1*). Atypical includes carriers of variants in *ATP13A2, RAB39B, SLC20A2,* and *WDR45*. *LRRK2* risk variants are R1628P and G2385R. (B) Global map of clinical trial locations recruiting gene variant carriers (*GBA1, LRRK2, PINK1,* and *PRKN*), obtained from the clinical trial registry https://clinicaltrials.gov/. Only one pin per gene and country is shown, regardless of the number of study sites within that country. AAC = African Admixed, AFR = African, AJ = Ashkenazi Jewish, AMR = Latinos and Indigenous people of the Americas, CAH = Complex Admixture, CAS = Central Asian, EAS = East Asian, EUR = European, FIN = Finnish, MDE = Middle Eastern, SAS = South Asian.

**Figure 5.**
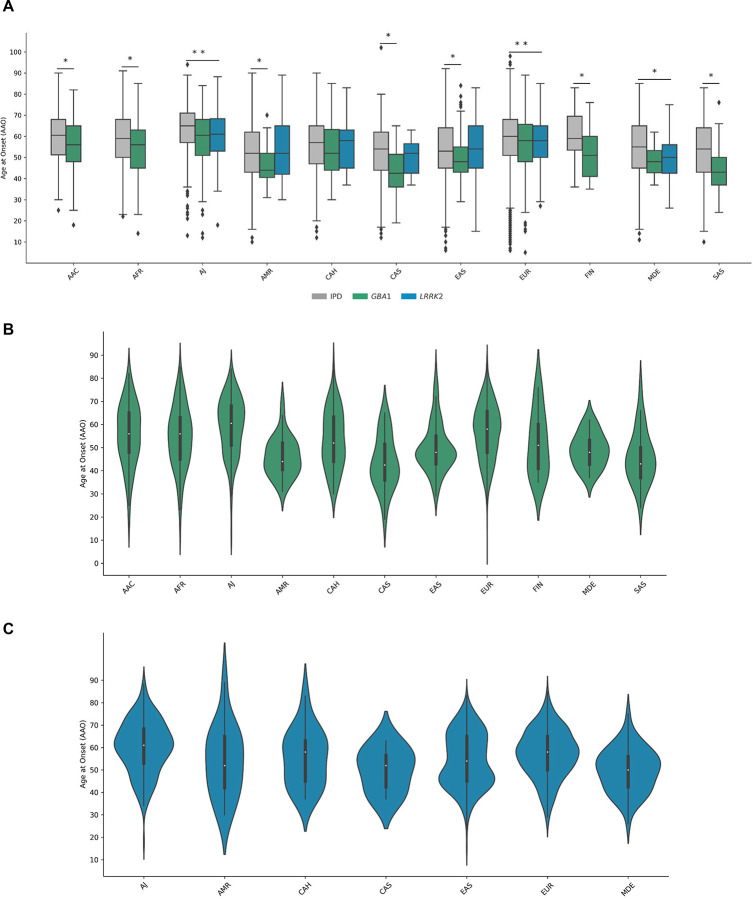
Ancestry-Stratified Age at Onset in Idiopathic, *GBA1*-, and *LRRK2*-associated Parkinson’s Disease. (A) Boxplots of ages at onset (AAO) for individuals with idiopathic Parkinson’s disease (IPD; gray), *GBA1*-associated PD (green), and *LRRK2*-linked PD (blue) grouped by ancestry. Boxes indicate interquartile ranges, horizontal lines represent median, and whiskers show the range. Outliers are plotted as individual points. Statistically significant group differences within ancestry based on linear regression are indicated by asterisks. (B) and (C) Violin plots of AAO for *GBA1*-associated PD (Panel B; green) and *LRRK2*-linked PD (Panel C; blue) across ancestries. Each violin reflects the distribution and median AAO within each ancestry group. AAC = African Admixed, AFR = African, AJ = Ashkenazi Jewish, AMR = Latinos and Indigenous people of the Americas, CAH = Complex Admixture, CAS = Central Asian, EAS = East Asian, EUR = European, FIN = Finnish, MDE = Middle Eastern, SAS = South Asian.

Among all affected variant carriers, we observed 53 distinct single nucleotide variants (SNVs) in 12 genes, next to structural variations like *SNCA* gene multiplications and variable *PRKN* exon deletions and duplications, two *LRRK2* risk variants, and 63 *GBA1* variants. *GBA1* variant carriers were most frequent overall and detected across all ancestries, albeit with substantially different variant spectra (Figure 2). *LRRK2* and *PRKN* variants were also identified across multiple ancestries, whereas causative variants in other genes were rarer and only identified in certain populations (Figure 3 and 4). Across multiple ancestries, individuals with *GBA1*-associated PD showed a significantly earlier median AAO of 3 to 8 years, compared to those with idiopathic PD (IPD) (Figure 5A and Supplementary Table 2). The AAO between *LRRK2*-PD and IPD differed significantly in AJ, EUR, and MDE, with *LRRK2*-PD showing a 1- to 4-year earlier onset (Figure 5A and Supplementary Table 2). Further, the AAO distribution among *GBA1* and *LRRK2* carriers showed heterogeneity across ancestries (Figure 5B and C).

The distribution of variants per gene across ancestries is summarized in Figures 3 and 4, Table 4, and Supplementary Table 3. Detailed results for each ancestry (excluding genetically enriched cohorts) are reported below, while results inclusive of genetically enriched cohorts are provided in Figure 3, Table 2, and Supplementary Table 3.

### African-Admixed ancestry (371 affected and 855 unaffected individuals)

Pathogenic or risk variants were observed in 35·6% of affected individuals (132/371). One early-onset PD patient (AAO=27 years) harbored a homozygous *PRKN* deletion. All other affected carriers harbored *GBA1* variants, most frequently the intronic rs3115534-G variant, found in 120/131 *GBA1* carriers (18 homozygous; overall frequency 32·3% [120/371]), while the remaining 11 individuals (3·0%) carried five distinct coding variants (Figure 2). *GBA1* variants were also identified in unaffected individuals (222/855; 26·0%), most commonly rs3115534-G (203/222 *GBA1* carriers; overall frequency 23·7% [203/855]). One unaffected individual harbored a *LRRK2* risk variant, and two pathogenic *LRRK2* variants, one being an unaffected family member (aged 27 years) of a PD patient (G2019S).

### African ancestry (1,030 affected and 1,743 unaffected individuals)

Among all identified affected carriers (536/1,030; 52·0%), *GBA1* variants were most frequent, particularly rs3115534-G (517/532 *GBA1* carriers; 123 homozygous; overall frequency 50·2% [517/1,030]). A small subset (15/1,030; 1·5%) carried nine distinct *GBA1* coding variants (Figure 2). Variants in other genes were rare and included *LRRK2* G2019S (n=1; 0·1%), *LRRK2* R1628P (n=2; 0·2%), and one (0·1%) early-onset PD patient (AAO=28 years) harboring two heterozygous *PRKN* variants. Among unaffected variant carriers (576/1,743; 33·0%), the majority harbored *GBA1* rs3115534-G (568/573; overall frequency 32·6% [568/1,743]) or *GBA1* coding variants (5/1,743; 0·3%). Three controls (0·2%), carried *LRRK2* variants (R1628P, n=2 and R1325Q, n=1).

### Ashkenazi Jewish ancestry (1,745 affected and 695 unaffected individuals)

In total, 27·6% (482/1,745) of affected individuals harbored pathogenic and risk variants, all in *GBA1* or *LRRK2*. 14·7% (256/1,745) carried eight distinct *GBA1* variants (Figure 2), most frequently N409S (178/256). Most *GBA1* carriers were PD patients, while a subset (n=12) had other neurodegenerative diseases. 11·5% (201/1,745) of affected individuals carried *LRRK2* variants, including one individual harboring R1067Q, while all others harbored G2019S. One *LRRK2* carrier was diagnosed with corticobasal degeneration, all others had PD. Another 25 individuals (1·4%) carried *LRRK2* G2019S and a *GBA1* variant. Among unaffected individuals, 8·6% (60/695) harbored *GBA1* or *LRRK2* variants.

### Latinos and Indigenous people of the Americas (2,140 affected and 1,481 unaffected individuals)

Among all identified affected carriers (134/2,140; 6·3%), *GBA1* variants were most frequent (75/2,140; 3·5%). Fifteen different *GBA1* variants were identified; E365K, T408M, and N409S were most common (Figure 2). 2·1% (46/2,140) carried *LRRK2* variants; all but one G2019S. One individual carried a G2019S and a *GBA1* variant. Ten individuals (0·5%) harbored biallelic *PRKN* variants (six homozygous and four likely compound heterozygous) and two (0·1%) biallelic *ATP13A2* variants. Among unaffected individuals, 0·8% (12/1,481) harbored *GBA1* and 0·1% (1/1,481) *LRRK2* variants.

### Complex Admixture (812 affected and 337 unaffected individuals)

Causative or risk variants were identified in 19·7% (160/812) of affected individuals, the majority of which were *GBA1* variants (127/812; 15·6%). Eight different *GBA1* variants were identified (Figure 2); rs3115534-G was most frequent (86/127 *GBA1* carriers; 4 homozygous; overall frequency 10·6% [86/812]). *LRRK2* variants were observed in 2·8% (23/812), mostly G2019S (17/23), and two carriers each of R1441C, R1628P, and G2385R. Two PD patients (0·25%) carried *VPS35* D620N, one (0·1%) harbored a *SNCA* multiplication, and seven early-onset patients (0·9%) harbored biallelic *PRKN* variants (four homozygous deletions and three likely compound heterozygous). Among unaffected carriers (42/337; 12·5%), *GBA1* variants were most frequent (36/337; 10·7%), particularly rs3115534-G (33/36 *GBA1* carriers; 2 homozygous; overall frequency 9·8% [33/337]). Six asymptomatic *LRRK2* carriers were identified (1·8%), three of which (all G2019S) were unaffected family members of PD patients (aged 45, 51, and 58 years).

### Central Asian ancestry (753 affected and 370 unaffected individuals)

We observed pathogenic or risk variants in 6·2% (47/753) of affected individuals, most frequently *GBA1* variants (30/753; 3·9%). Eight different *GBA1* variants were detected, most commonly E365K (n=11) and T408M (n=9) (Figure 2). We identified *LRRK2* variants in 1.6% (12/753); where the risk variants G2385R (n=8) and R1628P (n=3) were more frequent than pathogenic variants (R1441C, n=1). A subset (4/753; 0·5%) carried biallelic *PRKN* variants (three homozygous deletions and one likely compound heterozygous). Finally, twelve unaffected individuals (3·2%) carried *GBA1* (n=9) or *LRRK2* (n=3) risk variants.

### East Asian ancestry (3,363 affected and 2,503 unaffected individuals)

Amongst identified affected variant carriers (613/3,363; 18·2%), more than two-thirds carried one or both *LRRK2* risk variants R1628P and G2385R (439/3,363; 13·1%). Most were diagnosed with PD, while a subset (n=8) had atypical parkinsonism. Pathogenic *LRRK2* variants were rare (10/3,363; 0·3%) and included R1067Q, R1441C, R1441H, and G2019S. *GBA1* variants were observed in 3·4% (114/3,363), with a broad mutational spectrum including 29 distinct variants (Figure 2), most frequently L483P (33/114). 17 affected individuals (0·5%) harbored a risk variant in *LRRK2* and *GBA1*. Other dominantly inherited genes with identified variant carriers were *VPS35* D620N (n=3; 0·09%), *SNCA* (n=4; 0·1%), *SLC20A2* (n=2; 0·06%), and *DCTN1* (n=1; 0·03%). Additionally, 14 PD patients (0·4%) harbored biallelic *PRKN* variants (six homozygous deletions and six likely compound-heterozygous), and nine the homozygous *PINK1* L347P variant. Three additional individuals carried *PINK1* L347P and a *LRRK2* risk variant, and seven unaffected individuals harbored L347P in the heterozygous state. Additionally, *LRRK2* risk variants were identified in 8·7% (217/2,503) and *GBA1* variants in 0·8% (19/2,503) of unaffected individuals, and one control (0·04%) harbored *LRRK2* R1441C.

### European ancestry (28,859 affected and 19,539 unaffected individuals)

We identified pathogenic or risk variants across 11 genes in 10·1% (2,925/28,859) of affected individuals. Most frequent were *GBA1* variants (2,501/28,859; 8·7%), with 49 different identified variants (Figure 2), most commonly E365K (n=1,194) and T408M (n=755). 26 additional individuals (0·1%) were dual carriers of variants in *GBA1* and another gene, mostly *LRRK2*. Single *LRRK2* variants were observed in 0·9% (248/28,859). Eight different pathogenic variants were identified, most frequently G2019S (172/248), but also several other variants that were predominantly (R1325Q, R1441G/H/C) or exclusively (L1795F) identified in EUR individuals. We further identified carriers of *SNCA* multiplications (n=15) or coding variants (n=14) (29/28,859 in total, 0·1%), *VPS35* D620N (n=4, 0·01%), and *RAB32* S71R (n=7, 0·02%). In recessive genes, biallelic variants in *PRKN* were more frequent (63/28,859, 0·2%; 29 homozygous and 34 likely compound-heterozygous) than *PINK1* variants (n=8; 0·03%), and only a single biallelic *PARK7/DJ-1* E64D carrier was identified. Variants in atypical parkinsonism genes were observed in a subset (combined 7/28,859; 0·02%) and included *DCTN1* (n=4), *RAB39B* (n=2), and *WDR45* (n=1). All were diagnosed with PD. Finally, 5·1% (999/19,539) of unaffected individuals, including unaffected family members of PD patients, harbored variants in *GBA1* (n=936; 4·8%), especially E365K and T408M, *LRRK2* (n=54; 0·3%), *SNCA* (n=3; 0·02%), and *PRKN* (n=1; 0·01%).

### Finnish ancestry (120 affected and 14 unaffected individuals)

Only *GBA1* variants were identified in this ancestry. 11·7% of affected individuals (14/120) carried three different variants, E365K, T408M, and N409S (Figure 2), 13 diagnosed with PD and one with progressive supranuclear palsy (PSP). One unaffected individual (7·1%) harbored *GBA1* T408M.

### Middle Eastern ancestry (596 affected and 242 unaffected individuals)

Pathogenic or risk variants were identified in 13·4% (80/596) of affected individuals. Most frequent were pathogenic *LRRK2* variants (42/596; 7·0%), particularly G2019S (35/42), and a subset (n=7) with R1441C. All *LRRK2* variant carriers were diagnosed with PD. We identified 3·1% (19/596) harboring eleven different *GBA1* variants (Figure 2). One E365K carrier with PSP, while all other *GBA1* carriers were PD patients. Three additional PD patients (0·5%) harbored a pathogenic *LRRK2* and a *GBA1* variant. One PD patient carried *RAB32* S71R, and one a *SNCA* multiplication (0·2% each). A notable proportion of individuals carried biallelic variants in the recessive genes *PINK1* (n=10; 1·7%) and *PRKN* (n=4; 0·7%). One unaffected *LRRK2* G2019S carrier was identified (1/242; 0·4%).

### South Asian ancestry (499 affected and 235 unaffected individuals)

Pathogenic or risk variants were observed in 7·2% (36/499) of affected individuals, the majority of which were *GBA1* carriers (29/499; 5·8%). Except for one PSP patient, all others had PD. The most frequent *GBA1* variant was L483R (Figure 2), identified in 11 affected individuals and three unaffected family members of those (aged 40, 41, and 50 years, respectively). Further, one affected individual each carried a pathogenic (R1067Q) or *LRRK2* risk (R1628P) variant (total *LRRK2:* 0·4%), and five individuals harbored biallelic *PRKN* variants (5/499; 1·0%).

### Single heterozygous variants in recessively inherited genes

Across all ancestries, we identified 461 affected carriers of single heterozygous pathogenic variants in recessive genes, most frequently in *PRKN* (n=410), followed by *PINK1* (n=16) and *ATP13A2* (n=10), whereas variants in other genes were rare. Many carriers had an AAO ≤50 years (178/344, 51·7%; AAO missing for n=117). Three individuals carried two heterozygous *PRKN* variants but had an AAO >50 years, so we did not interpret them as likely compound heterozygous without further confirmation.

## Discussion

We performed a comprehensive analysis of causative and risk variants in PD-associated genes across diverse populations. Our work represents the largest ancestry-informed assessments to date, including >30% of non-European ancestry individuals, offering insights into the population-specific genetic architecture of PD. This is important as multiple ongoing or planned clinical trials target proteins encoded by PD-linked genes.^12,13^ Ensuring these advances are inclusive requires a detailed understanding of genetic variation beyond European ancestry.^14^

The overall yield of causal variants across affected individuals was ~2%, with substantial variations across ancestries ranging from <0·5% in FIN, AAC, and AFR to >10% in MDE and AJ. Including risk variants raised the yield to ~13%, aligning with prior studies.^15,16^ This increase was largely driven by *GBA1*, except in EAS, where the *LRRK2* risk variants R1628P and G2385R dominated.

*GBA1* variants were most frequent overall and identified across all populations, notably with different variant spectra: N409S was the most common variant in AJ, while E365K and T408M were more frequent in EUR, FIN, and CAS, but rare in AFR and EAS. The variant spectrum in EAS was broad, but L483P appeared more frequent than in other populations. The intronic variant rs3115534-G was by far the most frequently observed in AAC, AFR, and CAH, as previously reported.^17^ These findings emphasize the importance of *GBA1* as a globally relevant therapeutic target, an important observation given that most trials targeting glucocerebrosidase only include EUR or AJ individuals.^4^

*LRRK2* variants also showed ancestral variability. G2019S was detected across multiple ancestries, with the highest frequencies in MDE (reflecting North African Berbers) and AJ.^18,19^ While G2019S was also the most frequent *LRRK2* variant in EUR, the mutational spectrum was notably broader and included R1441C, R1441G, and L1795F with suggested founder effects in European sub-populations.^20–23^ In contrast, G2019S was largely absent from Asian populations, where the risk variants R1628P and G2385R dominated,^24^ especially in EAS. *LRRK2* variants differ in their impact on kinase activity,^25^ which may inform clinical trial design and therapeutic targeting. Investigating these actionable variants globally is key, particularly given the ongoing LRRK2-targeted trials.

*SNCA* variants were overall rare but most frequently identified in EUR, with only sporadic carriers in other populations, in line with previous reports.^26^ While *SNCA* multiplications were identified in EUR, EAS, MDE, and CAH individuals, missense variants (almost exclusively A53T) were predominantly observed in EUR (almost 50% of all *SNCA* carriers).

Other known autosomal dominant forms of PD caused by *VPS35* D620N or *RAB32* S71R were rare. *RAB32* variants were predominantly detected in EUR individuals, while *VPS35* variant carriers were observed across three different populations, with a relatively higher proportion in EAS and CAH compared to EUR. Given the low sample size of some populations, especially with WGS data, these findings need cautious interpretation. Nonetheless, detecting *VPS35* and *RAB32* variants across multiple ancestries underscores the importance of continued investigation in larger, diverse cohorts.

Investigating recessive forms of PD across populations, we found *PRKN* to be the most frequently implicated gene, followed by *PINK1*, while only a single EUR *DJ-1/PARK7* carrier was observed. Biallelic *PRKN* variants were identified across nearly all populations, except AJ and FIN. In Asian populations (EAS, CAS, and SAS), *PRKN* variants were more frequent than pathogenic *LRRK2* variants, although this warrants cautious interpretation given the small sample sizes in some groups. SNVs and CNVs in *PRKN* were observed at similar frequencies; however, CNVs appeared more frequent in EAS and SNVs in EUR. Identifying *PRKN* carriers across multiple ancestries reinforces its global relevance, as preclinical efforts increasingly explore therapeutic strategies targeting Parkin (encoded by *PRKN*) or its associated pathways.^12^ While *PINK1* variants were rare, we identified a notable proportion of MDE and EAS carriers. All EAS carriers harbored L347P, a variant known to be enriched in this population.^27^ We further identified numerous individuals carrying single heterozygous pathogenic variants in recessive PD genes. While these are not causative, many carriers had an early AAO ≤50 years, suggesting a second pathogenic variant, particularly a complex structural variant not captured by genotyping or short-read sequencing.

### Limitations

Our study focused on variants in known PD genes, primarily identified in European populations. This and the predominance of European-ancestry data in resources like ClinVar may bias variant interpretation in non-European groups. While our study allowed for a large-scale, clinically relevant assessment of those known variants, it limits the discovery of novel PD genes and variants that may be more relevant in other populations. Systematically studying large, well-powered non-European ancestry cohorts using WGS-rich datasets will be important to capture the full global spectrum of novel variation. Another important area will be investigating variants of uncertain significance in known PD genes. Some variants may be pathogenic in specific populations but are currently classified as of uncertain significance due to limited ancestry-specific reference data and low statistical power. Ancestry-aware (re)classification using variant frequency, functional, and segregation data will be key for establishing pathogenicity, improving genetic counseling and enabling trial access.^23,28^

While our study is large and ancestrally diverse, some populations were of limited sample size. This may inflate the relative contribution of known genes in well-powered populations and is particularly relevant for groups with limited WGS data, since select variants are not captured by genotyping (e.g., *RAB32* S71R and select *GBA1* variants).

The detection of compound-heterozygous variants was limited by the absence of phased data. We conservatively considered carriers of two heterozygous pathogenic variants in recessive genes with an AAO ≤50 years as likely compound heterozygous, but validation is needed.

CNV analyses did not include all investigated samples, likely resulting in an underestimation of *SNCA* and *PRKN* carriers. Lastly, findings were generated in a research setting; while we employed quality control measures and WGS validation where possible, further confirmation is needed.

### Conclusion

In conclusion, this is the largest study to date assessing the genetic spectrum of PD at a global level, offering crucial insights into the population-specific genetic architecture of PD and underscoring the critical need to expand PD genetics research beyond European ancestry. Ancestry-informed analysis improves diagnostic accuracy and risk interpretation and is essential to ensure equitable participation in clinical trials and access to emerging precision therapies. Addressing current gaps in data diversity is fundamental to fulfilling the promise of precision neurology on a global scale.

## Supplementary Material

Supplement 1

## Figures and Tables

**Table 1.** Study sample characteristics. Summary of sample numbers and cohort-level data by study type, including sex, age, age at onset (if applicable), and family history of Parkinson’s disease.

Group	n samples	Male (%)	Median Age* (range)	Median AAO (range)	Positive FH of PD** (%)
**Affected individuals**	41139	24886 (60.5)	67.5 (12–114), [unk for n = 10639]	59 (5–102), [unk for n = 13998]	8414 (20.5), [unk for n = 13635]
PD cohort unselected	33329	22718 (61.2)	68 (18–114), [unk for n = 6822]	60 (5–98), [unk for n = 14067]	6189 (18.6), [unk for n = 9840]
Monogenic recruitment	3421	1977 (57.8)	59 (19–101), [unk for n = 1211]	48 (6–102), [unk for n = 173]	1720 (50.3), [unk for n = 456]
Other phenotypes	3539	1971 (55.7)	72 (12–97), [unk for n = 2604]	66 (6–88), [unk for n = 2191]	184 (5.2), [unk for n = 3079]
Genetic enrichment affected	850	448 (77.2)	67 (21–91), [unk for n = 2]	57 (16–88), [unk for n = 454]	321 (37.8), [unk for n = 260]
**Unaffected individuals**	28742	14833 (51.6)	63 (16–104), [unk for n = 6706]	NA	1410 (4.9), [unk for n = 20193]
Controls	18585	9845 (53.0)	63 (17–104), [unk for n = 6672]	NA	689 (3.7), [unk for n = 10838]
Unaffected family members	184	92 (50.0)	71 (67–84), [unk for n = 0]	NA	184 (100), [unk for n = 0]
Population cohort	9245	4607 (49.8)	63 (45–94), [unk for n = 0]	NA	0 (0), [unk for n = 9245]
Genetic enrichment unaffected	728	289 (39.7)	59 (19–90), [unk for n = 2]	NA	537 (73.8), [unk for n = 110]
**TOTAL**	**73710**	**41947 (56.9)**		**NA**	**10524 (14.3), [unk for n = 34305]**

* Age refers to the age at recruitment/age at sample collection;

** Including related individuals.

AAO = Age at onset, FH = Family history, NA = not available/not applicable, PD = Parkinson's disease, unk = unknown, PD cohort unselected = Samples of PD cases recruited without specific selection criteria. Monogenic recruitment = Samples of affected individuals with a suspected monogenic cause of PD based on an early age at onset (≤50 years) or a positive family history of PD. Other phenotypes = Individuals with diagnoses other than PD, including atypical parkinsonism (e.g., dementia with Lewy bodies, corticobasal degeneration/syndrome, multisystem atrophy, and progressive supranuclear palsy), mild cognitive impairment and dementia (e.g., Alzheimer's disease, unspecified dementia), clinically diagnosed PD without a dopaminergic deficit in imaging (SWEDD), tremor, any other neurodegenerative diseases, and unspecified parkinsonism. Genetic enrichment affected = Individuals specifically recruited based on their genetic status, i.e., clinically affected *GBA1*, *LRRK2*, or *SNCA* variant carriers. Controls = Neurologically healthy controls. Unaffected family members = Unaffected family members of individuals with PD. Population cohort = Group representative of a general population, not affected by neurodegenerative diseases. Genetic enrichment unaffected = Individuals specifically recruited based on their genetic status, i.e., clinically unaffected *GBA1*, *LRRK2*, or *SNCA* variant carriers.

**Table 2.** Yield of genetic findings in affected individuals across ancestries. Proportion of affected individuals with genetic findings identified in this study, shown with and without genetically enriched cohorts, stratified by ancestry and by variant type (causal and risk variants vs. only causal variants).

	AAC	AFR	AJ	AMR	CAH	CAS	EAS	EUR	FIN	MDE	SAS	Total
**All affected individuals (incl. genetically enriched studies)**												
Samples investigated [n]	373	1030	2045	2143	813	756	3394	29339	121	620	505	**41139**
**Causal and risk variants**												
Genetic finding [n]	134	536	781	134	161	48	624	3262	14	104	38	5836
Yield [%]	35.92	52.04	38.19	6.25	19.80	6.34	18.39	11.12	11.57	16.77	7.52	14.19
**Only causal variants**												
Genetic finding [n]	1	2	425	59	30	6	53	621	0	85	6	1288
Yield [%]	0.27	0.19	20.78	2.75	3.69	0.79	1.56	2.12	0	13.71	1.19	3.13
**All affected individuals (excl. genetically enriched studies)**												
Samples investigated [n]	371	1030	1745	2140	812	753	3363	28859	120	596	499	40288
**Causal and risk variants**												
Genetic finding [n]	132	536	482	132	160	47	613	2925	14	80	36	5157
Yield [%]	35.58	52.04	27.62	6.17	19.70	6.24	18.23	10.14	11.67	13.42	7.21	12.80
**Only causal variants**												
Genetic finding [n]	1	2	226	57	29	5	46	393	0	61	6	826
Yield [%]	0.27	0.19	12.95	2.66	3.57	0.66	1.37	1.36	0	10.23	1.20	2.05

AAC = African Admixed, AFR = African, AJ = Ashkenazi Jewish, AMR = Latino and Indigenous people of the Americas, CAH = Complex Admixture, CAS = Central Asian, EAS = East Asian, EUR = European, FIN = Finnish, MDE = Middle Eastern, SAS = South Asian.

**Table 3.** Yield of genetic findings in unaffected individuals across ancestries. Proportion of unaffected individuals with genetic findings identified in this study, shown with and without genetically enriched cohorts, stratified by ancestry and by variant type (causal and risk variants vs. only causal variants).

	AAC	AFR	AJ	AMR	CAH	CAS	EAS	EUR	FIN	MDE	SAS	Total
**All unaffected individuals (incl. genetically enriched studies)**												
Samples investigated [n]	855	1743	1158	1485	339	371	2503	19795	14	244	235	**28742**
**Causal and risk variants**												
Genetic finding [n]	225	576	516	17	43	13	238	1233	1	3	3	2858
Yield [%]	26.32	33.04	44.56	1.14	12.68	3.50	9.51	6.23	7.14	1.23	1.33	9.94
**Only causal variants**												
Genetic finding [n]	1	1	258	3	5	1	1	216	0	3	0	497
Yield [%]	0.12	0.06	22.28	0.20	1.47	0.27	0.04	1.09	0	1.23	0	1.73
**All unaffected individuals (excl. genetically enriched studies)**												
Samples investigated [n]	855	1743	695	1481	337	370	2503	19539	14	242	235	28014
**Causal and risk variants**												
Genetic finding [n]	225	576	60	13	42	12	238	999	1	1	3	2170
Yield [%]	26.32	33.04	8.63	0.88	12.46	3.24	9.51	5.11	7.14	0.41	1.33	7.75
**Only causal variants**												
Genetic finding [n]	1	1	16	1	5	0	1	43	0	1	0	67
Yield [%]	0.12	0.06	2.30	0.07	1.48	0	0.04	0.22	0	0.41	0	0.24

AAC = African Admixed, AFR = African, AJ = Ashkenazi Jewish, AMR = Latino and Indigenous people of the Americas, CAH = Complex Admixture, CAS = Central Asian, EAS = East Asian, EUR = European, FIN = Finnish, MDE = Middle Eastern, SAS = South Asian.

**Table 4.** Summary of genetic findings across all ancestries. Overview of variant findings per gene by ancestry, including affected and unaffected individuals, limited to individuals from non-genetically enriched cohorts.

		PD risk	Typical autosomal-dominant PD					Early-onset recessive PD			Atypical parkinsonism					*Dual carriers* **
		*GBA1*	*LRRK2*	*LRRK2 risk* *	*SNCA*	*VPS35*	*RAB32*	*PINK1*	*PRKN*	*PARK7/DJ-1*	*ATP13A2*	*DCTN1*	*SLC20A2*	*RAB39B*	*WDR45*	
**AAC**	Affected	131	0	0	0	0	0	0	1	0	0	0	0	0	0	0
	Unaffected	222	2	1	0	0	0	0	0	0	0	0	0	0	0	0
**AFR**	Affected	532	1	2	0	0	0	0	1	0	0	0	0	0	0	0
	Unaffected	573	1	2	0	0	0	0	0	0	0	0	0	0	0	0
**AJ**	Affected	256	201	0	0	0	0	0	0	0	0	0	0	0	0	25
	Unaffected	44	15	0	0	0	0	0	0	0	0	0	0	0	0	1
**AMR**	Affected	75	46	0	0	0	0	0	10	0	2	0	0	0	0	1
	Unaffected	12	1	0	0	0	0	0	0	0	0	0	0	0	0	0
**CAH**	Affected	127	19	4	1	2	0	0	7	0	0	0	0	0	0	0
	Unaffected	36	5	1	0	0	0	0	0	0	0	0	0	0	0	0
**CAS**	Affected	30	1	11	0	0	0	0	4	0	0	0	0	0	0	1
	Unaffected	9	0	3	0	0	0	0	0	0	0	0	0	0	0	0
**EAS**	Affected	114	10	439	4	3	0	9	14	0	0	1	2	0	0	17
	Unaffected	19	1	217	0	0	0	0	0	0	0	0	0	0	0	1
**EUR**	Affected	2501	248	31	29	4	7	8	63	1	0	4	0	2	1	26
	Unaffected	936	35	19	3	0	0	0	1	0	0	0	0	0	0	5
**FIN**	Affected	14	0	0	0	0	0	0	0	0	0	0	0	0	0	0
	Unaffected	1	0	0	0	0	0	0	0	0	0	0	0	0	0	0
**MDE**	Affected	19	42	0	1	0	1	10	4	0	0	0	0	0	0	3
	Unaffected	0	1	0	0	0	0	0	0	0	0	0	0	0	0	0
**SAS**	Affected	29	1	1	0	0	0	0	5	0	0	0	0	0	0	0
	Unaffected	3	0	0	0	0	0	0	0	0	0	0	0	0	0	0

* Variants identified as *LRRK2* PD risk variants in the Asian population, including rs33949390 (chr12:40320043:G:C, p.R1628P) and rs34778348 (chr12:40363526:G:A, p.G2385R).

** Dual carriers refers to carriers of two pathogenic/likely pathogenic or PD risk variants in two different genes.

AAC = African Admixed, AFR = African, AJ = Ashkenazi Jewish, AMR = Latino and Indigenous people of the Americas, CAH = Complex Admixture, CAS = Central Asian, EAS = East Asian, EUR = European, FIN = Finnish, MDE = Middle Eastern, SAS = South Asian.

## Data Availability

Data used in the preparation of this article were obtained from the Global Parkinson’s Genetics Program (GP2; https://gp2.org). Specifically, we used Tier 2 data from GP2 releases 8 (DOI 10.5281/zenodo.13755496) and 9 (DOI 10.5281/zenodo.14510099). Tier 1 data can be accessed by completing a form on the Accelerating Medicines Partnership in Parkinson’s Disease (AMP^®^-PD) website (https://amp-pd.org/register-for-amp-pd). Tier 2 data access requires approval and a Data Use Agreement signed by your institution. Qualified researchers are encouraged to apply for direct access to the data through AMP PD. All code generated for this article, and the identifiers for all software programs and packages used, are available on GitHub (https://github.com/GP2code/GP2-global-genetic-variant-landscape) and were given a persistent identifier via Zenodo (DOI 10.5281/zenodo.15699539). A detailed list of all identified variant carriers, including corresponding anonymised GP2-IDs, variant details as well as basic demographic and clinical characteristics are available to qualified researchers and upon reasonable request from the corresponding author.

## Putting research into context

### Evidence before this study

Genetic discoveries have drastically advanced our understanding of Parkinson’s disease (PD), including the identification of rare monogenic causes and common risk variants. However, according to our PubMed-based (https://pubmed.ncbi.nlm.nih.gov/) literature review, the majority of genetic data has been generated in PD cohorts of European ancestry. The sample sets of two recent comprehensive, large-scale genetic screening studies, the PD GENEration North America and the Rostock International Parkinson’s disease (ROPAD) study, comprised 85% and 96% of White participants, respectively. Moreover, a global survey of monogenic PD, published in 2023, reported that 91% of genetically confirmed individuals with PD were of European ancestry, underscoring the major gap in ancestral representation in PD genetics research. Although ancestry-specific variants and founder effects have been reported for select PD-associated genes such as *GBA1*, *LRRK2*, and *PINK1*, comprehensive, globally- and ancestry-representative studies are missing, thereby constraining the applicability of genetic diagnostics and the development and inclusivity of equitable, ancestry-informed therapeutic strategies.

### Added value of this study

This study, conducted within the framework of the Global Parkinson’s Genetics Program (GP2), represents the largest multi-ancestry exploratory genetic investigation of PD to date, including nearly 70,000 individuals from around the world, with more than 20,000 participants of non-European ancestry (representing ~30% of the study cohort). By exploring the genetic landscape of pathogenic variants in established PD-linked genes and PD risk-associated variants across 11 diverse genetically defined ancestry groups, it provides crucial insights into the global genetic landscape of PD. This work highlights both shared and ancestry-specific contributions and emphasizes the need to consider genetic diversity in research and clinical practice. Our findings highlight the importance of *GBA1* as a globally relevant genetic contributor to PD risk, with variant carriers identified across all investigated populations, reinforcing it as a relevant therapeutic target across ancestries. Similarly, *LRRK2* variant carriers, currently already targeted in clinical trials, and individuals with *PRKN* alterations, increasingly being explored as a therapeutic target in preclinical studies, were identified across multiple ancestral groups.

However, overall, investigating variants in known PD-linked genes resulted in substantially different yields across ancestries, underscoring the limited applicability of current PD gene and variant panels across ancestries and suggesting yet unexplored population-specific variations, especially in currently underrepresented populations, where newly identified variants in established PD-genes often remain of uncertain significance due to limited sample size and reference data.

### Implications of all the available evidence

As precision medicine approaches are increasingly integrated into PD research and clinical trials, it is critical to ensure that emerging therapies are applicable and accessible to individuals of all ancestral backgrounds. Current trials targeting gene variant carriers predominantly enroll participants of European or Ashkenazi Jewish ancestry, raising concerns about equity and efficacy in a global multi-ancestry context. This study underscores the importance of globally inclusive genetic efforts like GP2 to close these existing gaps, improve diagnostic equity, and enable more representative and effective implementation of precision neurology worldwide.
