# Supplementary material for: The Global Landscape of Genetic Variation in Parkinson’s disease: Multi-Ancestry Insights into Established Disease Genes and their Translational Relevance
Source: medRxiv. 2025 Jul 11:2025.07.08.25330815. Preprint. [Version 1] doi: 10.1101/2025.07.08.25330815 (PMC12265751; doi:10.1101/2025.07.08.25330815)
Supplement: Supplement 1 [file media-1.pdf]

Supplementary Material

The Global Landscape of Genetic Variation in Parkinson's disease: Multi-Ancestry Insights into Established Disease Genes and their Translational Relevance

Lara M. Lange, MD,[1,2] Zih-Hua Fang, PhD,[3] Mary B. Makarious, PhD,[4,5] Nicole Kuznetsov, MPS,[4,5] Kajsa Atterling Brolin, PhD,[6,7] Shannon Ballard, PhD,[4,5] Soraya Bardien, PhD,[8] Maria Leila Doquenia, MD,[9,10,11] Peter Heutink, PhD,[12] Henry Houlden, MD,[13] Hirotaka Iwaki, PhD,[4,5] Simona Jasaityte, MSc,[14] Lietsel Jones, MSc,[4,5] Johanna Junker, MD,[15,16] Rauan Kaiyrzhanov, MD,[13,17] Mathew J. Koretsky, BSc,[4,5] Kishore R. Kumar, PhD,[18,19,20] the Latin American Research Consortium on the Genetics of Parkinson's Disease (LARGE-PD), Hampton L. Leonard, MSc,[4,5] Kristin S. Levine, MSc,[4,5] Shen-Yang Lim, MD,[21] Niccoló E. Mencacci, MD,[22] Wael M. Y. Mohamed, PhD,[23,24] Mike A. Nalls, PhD,[4,5] Alastair J. Noyce, MD,[7] Rajeev Ojha, MD,[25] Njideka U. Okubadejo, MD,[26] Shoaib ur Rehman, PhD,[27] Laurel Screven, PhD, [12] Chingiz Shashkin, MD,[28,29] Sophia Sopromadze, MD,[30,31] Eleanor J. Stafford, MSc,[14] Ai Huey Tan, MD,[21] Manuela Tan, PhD,[7,32] Zaruhi Tavadyan, MD,[33] Joanne Trinh, PhD,[2] Bayasgalan Tserensodnom, MD,[34] Enza Maria Valente, MD,[35,36] Dan Vitale, MSc,[4,5] Nazira Zharkinbekova, MD,[18] Katja Lohmann, PhD,[2] Sara Bandres-Ciga, PhD,[5] Cornelis Blauwendraat, PhD,[12] Andrew Singleton, PhD,[12] Huw R. Morris, PhD,[14,37,#] Christine Klein, MD,[2#] and the Global Parkinson's Genetics Program (GP2)

[1] Laboratory of Neurogenetics, National Institute on Aging, Bethesda, Maryland, USA
[2] Institute of Neurogenetics, University of Luebeck, Luebeck, Germany
[3] German Center for Neurodegenerative Diseases (DZNE), Tübingen, Germany
[4] DataTecnica, Washington, DC, USA
[5] Center for Alzheimer's and Related Dementias (CARD), National Institute on Aging and National Institute of Neurological Disorders and Stroke, National Institutes of Health, Bethesda, MD, USA
[6] Translational Neurogenetics Unit, Department of Experimental Medical Science, Lund University, Lund, Sweden
[7] Centre for Preventive Neurology, Wolfson Institute of Population Health, Queen Mary University of London, London, United Kingdom
[8] Division of Molecular Biology and Human Genetics, Faculty of Medicine and Health Sciences, Stellenbosch University; South African Medical Research Council/Stellenbosch University Genomics of Brain Disorders Research Unit, Cape Town, South Africa
[9] Department of Neuroscience and Brain Health, Metropolitan Medical Center, Manila, Philippines
[10] Department of Clinical Neurosciences, Mary Mediatrix Medical Center, Lipa, Batangas, Philippines
[11] Department of Anatomy, University of the East Ramon Magsaysay Memorial Medical Center, Quezon City, Philippines
[12] The Global Parkinson's Genetics Program (GP2)
[13] University College London, Institute of Neurology, Department of Neuromuscular Diseases, Queen Square, WC1N 3BG London, UK
[14] Department of Clinical and Movement Neurosciences, UCL Queen Square Institute of Neurology, London, UK
[15] University Hospital Schleswig Holstein, Campus Luebeck, Luebeck, Germany
[16] Center for Movement Disorders and Neuromodulation, University Hospital Duesseldorf, Duesseldorf, Germany
[17] Department of Neurology, South Kazakhstan Medical Academy, 160019, Shymkent, Kazakhstan
[18] Neurology Department and Molecular Medicine Laboratory, Concord Repatriation General Hospital and University of Sydney, Concord, NSW, 2139
[19] Translational Neurogenomics Group, Genomics and Inherited Disease Program, The Garvan Institute of Medical Research, Darlinghurst, NSW, 2010, Australia.
[20] Faculty of Medicine and Health, University of New South Wales, Sydney, NSW, Australia
[21] Division of Neurology, Department of Medicine, Faculty of Medicine, University of Malaya, Kuala Lumpur, Malaysia
[22] Department of Neurology, Northwestern University Feinberg School of Medicine, Chicago, IL, USA
[23] Department of Basic Medical Sciences, Kulliyyah of Medicine, International Islamic University Malaysia (IIUM), Kuantan, Pahang, Malaysia
[24] Clinical Pharmacology Department, Menoufia Medical School, Menoufia University, Shebin El-Kom, Menoufia, Egypt

[25] Department of Neurology, Tribhuvan University Teaching Hospital, Kathmandu, Nepal
[26] Neurology Unit, Department of Medicine, College of Medicine, University of Lagos, Lagos State, Nigeria
[27] Department of Biotechnology, University of science and Technology Bannu, Pakistan
[28] International Research Institute of Postgraduate Education, Department of Neurosurgery and Neurology, Almaty, Kazakhstan
[29] Shashkin Clinic, Almaty, Kazakhstan
[30] Department of Neurology, Ivane Javakhishvili Tbilisi State University, Tbilisi, Georgia
[31] East European University (EEU), Tbilisi, Georgia
[32] Department of Neurology, Oslo University Hospital, Oslo, Norway
[33] National Institute of Health, Yerevan, Armenia
[34] Department of Neurology, School of Medicine, Mongolian National University of Medical Sciences, Ulaanbaatar, Mongolia
[35] Department of Molecular Medicine, University of Pavia, Pavia, Italy
[36] Neurogenetics Research Center, IRCCS Mondino Foundation, Pavia, Italy
[37] UCL Movement Disorders Centre, University College London, London, UK

[#] Shared last authors

Correspondence to
Lara M. Lange
Laboratory of Neurogenetics
National Institute on Aging (NIA), National Institutes of Health (NIH)
9000 Rockville Pike
Bethesda, MD 20892,
United States of America
Email: lara.lange@nih.gov

or Christine Klein
Institute of Neurogenetics
University of Luebeck
Ratzeburger Allee 160
23538 Luebeck,
Germany
Email: christine.klein@uni-luebeck.de
Phone: +49-451-3101-8200

Table of Content

	Page
**Supplementary Methods**	
Ethics declaration	4
Whole-genome sequencing (WGS) data (GP2 and AMP-PD) processing	4
Clinical exome sequencing data (PDGENEration) processing	4
Genome-wide genotyping data from the NeuroBooster Array (NBA) processing	4-5
Variants of interest and pathogenicity evaluation	5
Validation of genetic findings and concordance check	5
Additional statistical analyses	5
**Supplementary Results**	
Validation of genetic findings and concordance check	6
**Supplementary References**	7
**Supplementary Figures**	
Supp. Figure 1: Global allele frequencies of selected common *GBA1* and *LRRK2* variants.	8-9
Supp. Figure 2: Cluster plots	10-20
**Supplementary Tables**	
Supp. Table 1: Overview of included samples investigated in this study.	21
Supp. Table 2: Results of linear regression analysis comparing age at onset (AAO) between idiopathic PD (IPD, reference group) and *LRRK2*- and *GBA1*-associated PD across ancestries.	22
Supp. Table 3: Summary of genetic findings across all ancestries.	23-26
Supp. Table 4: List of all identified variants and concordance check NBA and CES/WGS.	27-36

Supplementary Methods

Ethics declaration

This study was approved by ethics committees or institutional review boards of all participating sites and conducted in accordance with their ethical standards. Informed consent for study participation was obtained from all participants.

Whole-genome sequencing (WGS) data (GP2 and AMP-PD) processing

We used WGS data generated as part of GP2 Data Release 8 (DOI 10.5281/zenodo.13755496). This release included a total of 7,734 participants. All samples were genome sequenced to an average of 30x coverage with 150bp paired-end reads following Illumina's TruSeq PCR-free library preparation protocol. We followed AMP-PD's functional equivalence pipeline[1] to produce the sequence alignment against the GRCh38DH reference genome. DeepVariant v.1.6.1[2] (https://github.com/google/deepvariant) was used to generate the single-sample variant calls, and joint-genotyping was performed using GLnexus v1.4.3 (https://github.com/dnanexus-rnd/GLnexus) with the preset DeepVariant WGS configuration[3]. Genotypes were set to be missing after variant quality control defined as genotype quality >=10, read depth >=10, and heterozygous allele balance between 0.2 and 0.8, and retained high-quality variants with a call rate > 0.95 after quality control. Genetic ancestry was determined using GenoTools v1.2.3 (https://github.com/GP2code/GenoTools) using default settings[4].
Variants in the genes of interest were extracted using PLINK[5,6] and annotated with ANNOVAR[7,8]. We used the Gauchian pipeline (https://github.com/Illumina/Gauchian)[9] for WGS data as a variant caller for *GBA1*.

Clinical exome sequencing data (PDGENEration) processing

We used clinical exome sequencing data from 10,454 individuals with PD generated by PDGENEration[10] and released as part of GP2 Data Release 8 (DOI 10.5281/zenodo.13755496)[11]. Data processing followed the same pipeline we used for WGS data (described above). We performed joint-genotyping using GLnexus v1.4.3 with the preset DeepVariant whole exome sequencing (WES) configuration and followed the same criteria for sample and variant quality used for WGS data.
Variants in the genes of interest were extracted using PLINK[5,6] and annotated with ANNOVAR[7,8].

Genome-wide genotyping data from the NeuroBooster Array (NBA) processing

We used raw genotyping data generated as part of GP2 Data Release 9 (DOI 10.5281/zenodo.14510099). This release included a total of 68,249 individuals from eleven different ancestries. Genotyping was performed using the NeuroBooster Array (NBA; v.1.0, Illumina, San Diego, CA)[12]. Raw genotyping data underwent quality control and genetic ancestry prediction using GenoTools v1.2.3 with the default settings[4].
Variants in the genes of interest were extracted using PLINK[5,6] and annotated with ANNOVAR[7,8]. Copy number variation (CNV) analyses were performed for 44,280 samples using genotyping data as described before[13] and included screening for *SNCA* multiplications and *PRKN* deletions and duplications.

Further, we imputed rs3115534-G using the Michigan Imputation Server 2 (https://imputationserver.sph.umich.edu/; accessed April 2025)[14] by uploading chromosome 1 of each ancestry separately (GP2 release 9; 10.5281/zenodo.14510099). All ancestries were imputed to the 1000G Phase 3 30x (GRCh38/hg38) panel. Below is a table outlining the allele frequencies (AF), minor allele frequencies (MAF), average concordance between imputed genotypes and the true genotypes (AVG_CS), and the estimated squared correlation between the true allele dosage and the imputed allele dosage (R^2) for rs3115534 per ancestry. We considered R^2 scores >0.90 reliable, thus, only variant carriers of African Admixed (AAC), African (AFR), and Complex Admixture (CAH) ancestry were extracted.

Ancestry	AF	MAF	AVG_CS	R2
AAC	0.855094	0.144906	0.993595	0.960604
AFR	0.771176	0.228824	0.993347	0.970107
AJ	0.994816	0.00518388	0.99497	0.069857
AMR	0.983785	0.0162153	0.991627	0.507223
CAH	0.936112	0.0638879	0.993281	0.904852
CAS	0.991213	0.00878704	0.991213	0.101378

**EAS**	0.9916	0.00839955	0.991825	0.0730985
**EUR**	0.992499	0.00750059	0.993165	0.195994
**FIN**	0.994379	0.00562149	0.994379	0.032264
**MDE**	0.982031	0.0179694	0.993657	0.667746
**SAS**	0.991229	0.0087713	0.991714	0.0938885

AAC = African admixed ancestry, AFR = African ancestry, AJ = Ashkenazi Jewish, AMR = Latinos and Indigenous People of the Americas, CAH = Complex admixture ancestry, CAS = Central Asian ancestry, EAS = East Asian ancestry, EUR = European ancestry, FIN = Finnish ancestry, MDE = Middle Eastern ancestry, SAS = South Asian ancestry

Variants of interest and pathogenicity evaluation

We focused our analyses on variants predicted as pathogenic/likely pathogenic according to ClinVar (https://www.ncbi.nlm.nih.gov/clinvar/)[15] and/or the consensus recommendations of the American College of Medical Genetics and Genomics (ACMG)[16]. For variants with conflicting ClinVar predictions or those absent from ClinVar, pathogenicity was evaluated using Franklin (https://franklin.genoox.com) and Varsome (https://varsome.com/)[17], both based on the ACMG criteria. We added *RAB32* variant (chr6:146544084:C:G, p.S71R), that is not yet included in ClinVar and still predicted to be a variant of uncertain significance despite convincing evidence of a causal role from the literature.[18–21] We further included more common *LRRK2* variants classified as PD risk-associated based on association studies, including rs33949390 (chr12:40320043:G:C, p.R1628P) and rs34778348 (chr12:40363526:G:A, p.G2385R), given their translational relevance. Individuals harboring two pathogenic/likely pathogenic variants in recessive genes with an age at onset (AAO) ≤50 years were considered likely compound heterozygous although the phase could not be determined. In addition to causal genes, we also investigated *GBA1* variants, given their substantial translational impact. For the purpose of this study and in the context of PD, all *GBA1* variants mentioned in the main text are referred to as risk variants (including those predicted to be causal for Gaucher's disease); including variants of different severities (i.e., severe, mild, and risk). However, the different variant severities were evaluated with the *GBA1*-PD browser (https://pdgenetics.shinyapps.io/gba1browser/)[22] and their distribution is provided in Figure 2.

Validation of genetic findings

Performing wet-lab or CLIA-certified validation of genetic findings was out of the scope of this study. We performed a concordance check, using WGS and CES data as a validation of genotyping findings for samples that underwent both analyses (Supplementary Table 4), especially for rare variants. Further, we generated cluster plots to evaluate and ensure adequate genotyping performance of identified variants (Supplementary Figure 2).

Additional statistical analyses

We performed a logistic linear regression to compare the ages at onset between individuals with idiopathic PD and individuals with either *LRRK2*-linked PD or *GBA1*-associated PD. We considered all individuals that did not carry a causative of high-risk variant in the genes of interest investigated in this study as "idiopathic". The linear regression was performed using idiopathic PD as the reference group; we adjusted our analysis for sex. A p-value of <0.05 was considered statistically significant.

Supplementary Results

Concordance between genotyping and WGS or CES data

Out of all identified carriers in this study, 2,804 out of 5,010 (56.0%) had both NBA genotyping and CES or WGS data available. Among 2,062 individuals with both NBA and WGS data, 377 variants were detected only by WGS, including frameshift and splicing variants that are inherently difficult to detect by genotyping, as well as a subset of variants that were identified through NBA genotyping in other individuals. Of the remaining carriers, 99.1% (1,670/1,685) had variants confirmed by both methods, while only 0.9% (15/1,685) of variants detected by NBA genotyping were not validated by WGS. The latter included variants that were independently validated in other samples. Similarly, among 742 individuals with both NBA and CES data, 122 variants were exclusively detected by CES. These included frameshift and splicing variants, as well as variants previously detected by NBA in other individuals. Among the remaining carriers, 96.6% (599/620) of variants were confirmed by both techniques, while 3.4% (21/620) of NBA-identified variants were not validated by CES.

Supplementary Table 4 provides detailed information on all variants identified in this study, including the number of carriers, the method of detection, and the percentage of confirmed findings among individuals with both NBA and sequencing data.

Supplementary References

1 Regier AA, Farjoun Y, Larson DE, *et al.* Functional equivalence of genome sequencing analysis pipelines enables harmonized variant calling across human genetics projects. *Nat Commun* 2018; **9**: 4038.
2 Poplin R, Chang P-C, Alexander D, *et al.* A universal SNP and small-indel variant caller using deep neural networks. *Nat Biotechnol* 2018; **36**: 983–7.
3 Yun T, Li H, Chang P-C, Lin MF, Carroll A, McLean CY. Accurate, scalable cohort variant calls using DeepVariant and GLnexus. *Bioinformatics* 2021; **36**: 5582–9.
4 Vitale D, Koretsky MJ, Kuznetsov N, *et al.* GenoTools: An open-source Python package for efficient genotype data quality control and analysis. *G3 (Bethesda)* 2024; published online Nov 20. DOI:10.1093/g3journal/jkae268.
5 Purcell S, Neale B, Todd-Brown K, *et al.* PLINK: a tool set for whole-genome association and population-based linkage analyses. *Am J Hum Genet* 2007; **81**: 559–75.
6 Chang CC, Chow CC, Tellier LC, Vattikuti S, Purcell SM, Lee JJ. Second-generation PLINK: rising to the challenge of larger and richer datasets. *Gigascience* 2015; **4**: 7.
7 Wang K, Li M, Hakonarson H. ANNOVAR: functional annotation of genetic variants from high-throughput sequencing data. *Nucleic Acids Res* 2010; **38**: e164.
8 Yang H, Wang K. Genomic variant annotation and prioritization with ANNOVAR and wANNOVAR. *Nat Protoc* 2015; **10**: 1556–66.
9 Toffoli M, Chen X, Sedlazeck FJ, *et al.* Comprehensive short and long read sequencing analysis for the Gaucher and Parkinson's disease-associated GBA gene. *Commun Biol* 2022; **5**: 670.
10 Cook L, Verbrugge J, Schwantes-An T-H, *et al.* Parkinson's disease variant detection and disclosure: PD GENEration, a North American study. *Brain* 2024; **147**: 2668–79.
11 Leonard H, Nalls M, Vitale D, *et al.* Global Parkinson's Genetics Program data release 7. 2024. DOI:10.5281/ZENODO.10962119.
12 Bandres-Ciga S, Faghri F, Majounie E, *et al.* NeuroBooster array: A genome-wide genotyping platform to study neurological disorders across diverse populations. *Mov Disord* 2024; published online Sept 16. DOI:10.1002/mds.29902.
13 Kuznetsov N, Daida K, Makarious MB, *et al.* CNV-Finder: Streamlining copy number variation discovery. bioRxivorg. 2024; published online Nov 23. DOI:10.1101/2024.11.22.624040.
14 Das S, Forer L, Schönherr S, *et al.* Next-generation genotype imputation service and methods. *Nat Genet* 2016; **48**: 1284–7.
15 Landrum MJ, Lee JM, Riley GR, *et al.* ClinVar: public archive of relationships among sequence variation and human phenotype. *Nucleic Acids Res* 2014; **42**: D980–5.
16 Richards S, Aziz N, Bale S, *et al.* Standards and guidelines for the interpretation of sequence variants: a joint consensus recommendation of the American College of Medical Genetics and Genomics and the Association for Molecular Pathology. *Genet Med* 2015; **17**: 405–24.
17 Kopanos C, Tsiolkas V, Kouris A, *et al.* VarSome: the human genomic variant search engine. *Bioinformatics* 2019; **35**: 1978–80.
18 Gustavsson EK, Follett J, Trinh J, *et al.* RAB32 Ser71Arg in autosomal dominant Parkinson's disease: linkage, association, and functional analyses. *Lancet Neurol* 2024; **23**: 603–14.
19 Monfrini E, Minardi R, Valzania F, *et al.* RAB32 mutation in Parkinson's disease. *Lancet Neurol* 2024; **23**: 961–2.
20 Beetz C, Radefeldt M, Tripolszki K, Kandaswamy KK, Bauer P, ROPAD Study Group. RAB32 mutation in Parkinson's disease. *Lancet Neurol* 2024; **23**: 961.
21 Cogan G, Tesson C, Brefel-Courbon C, *et al.* Confirmation of RAB32 Ser71Arg involvement in Parkinson's disease. *Mov Disord* 2025; **40**: 174–5.
22 Parlar SC, Grenn FP, Kim JJ, Baluwendraat C, Gan-Or Z. Classification of GBA1 variants in Parkinson's disease: The GBA1-PD browser. *Mov Disord* 2023; **38**: 489–95.

Supplementary Tables

Supplementary Table 1. Overview of included samples investigated in this study.

		AAC	AFR	AJ	AMR	CAH	CAS	EAS	EUR	FIN	MDE	SAS	Total
**NBA only**	**Total**	1038	2501	1650	3189	750	1023	4556	39792	106	711	479	**55795**
	**Affected**	193	822	968	1717	416	659	2082	20618	93	467	252	**28287**
	**Unaffected**	845	1679	682	1472	334	364	2474	19174	13	244	227	**27508**
**CES only**	**Total**	9	12	99	32	106	4	7	668	3	5	3	**948**
	**Affected**	9	12	99	32	106	4	7	668	3	5	3	**948**
	**Unaffected**	0	0	0	0	0	0	0	0	0	0	0	**0**
**WGS only**	**Total**	3	6	66	22	17	31	11	448	2	6	4	**616**
	**Affected**	2	5	46	19	15	31	11	309	1	6	4	**449**
	**Unaffected**	1	1	20	3	2	0	0	139	1	0	0	**167**
**NBA + CES**	**Total**	126	51	530	243	228	25	69	4114	14	36	62	**5498**
	**Affected**	126	51	530	243	228	25	69	4114	14	36	62	**5498**
	**Unaffected**	0	0	0	0	0	0	0	0	0	0	0	**0**
**NBA + WGS**	**Total**	51	203	834	132	51	41	1249	3935	9	105	187	**6797**
	**Affected**	42	140	378	122	48	34	1220	3453	9	105	179	**5730**
	**Unaffected**	9	63	456	10	3	7	29	482	0	0	8	**1067**
**CES + WGS**	**Total**	1	0	24	10	0	3	5	177	1	1	5	**227**
	**Affected**	1	0	24	10	0	3	5	177	1	1	5	**227**
	**Unaffected**	0	0	0	0	0	0	0	0	0	0	0	**0**
**Total**	**Total**	1228	2773	3203	3628	1152	1127	5897	49134	135	864	740	**69881**
	**Affected**	373	1030	2045	2143	813	756	3394	29339	121	620	505	**41139**
	**Unaffected**	855	1743	1158	1485	339	371	2503	19795	14	244	235	**28742**

The "Affected" group summarizes the number of samples of all affected individuals investigated in this study, including individuals from unselected PD cohorts, individuals submitted to the monogenic GP2 study arm (e.g., samples with an AAO ≤50 years and/or a positive family history of PD), individuals with other neurological and neurodegenerative phenotypes (e.g., atypical parkinsonism, different types of dementia, SWEDD, tremor, etc.) and affected individuals submitted as part of genetically enriched cohorts (e.g., known *GBA1* or *LRRK2* variant carriers). The "Unaffected" group summarizes the number of samples of all unaffected individuals investigated in this study, including healthy controls, unaffected family members of individuals with PD, individuals from population cohorts, and unaffected individuals submitted as part of genetically enriched cohorts (e.g., asymptomatic *GBA1* and *LRRK2* carrier).
AAC = African Admixed, AFR = African, AJ = Ashkenazi Jewish, AMR = Latino and Indigenous people of the Americas, CAH = Complex Admixture, CAS = Central Asian, CES = Clinical exome sequencing, EAS = East Asian, EUR = European, FIN = Finnish, MDE = Middle Eastern, NBA = NeuroBooster Array (genotyping), SAS = South Asian, WGS = short-read whole genome sequencing.

Supplementary Table 2. Results of linear regression analysis comparing age at onset (AAO) between idiopathic PD (IPD, reference group) and *LRRK2*- and *GBA1*-associated PD across ancestries.

Ancestry	Group	N	Coef.	Std.Err.	t	P	[0.025	0.975]
**AAC**	*GBA1*-PD compared to IPD	372	-3.7740807	1.7067289	-2.2112948	0.027889094	-7.1349125	-0.4132489
**AFR**	*GBA1*-PD compared to IPD	1026	-3.5604535	1.55789137	-2.2854312	0.023019332	-6.6268406	-0.4940664
**AJ**	*GBA1*-PD compared to IPD	2004	-4.1351982	0.7974728	-5.1853783	2.442E-07	-5.6994471	-2.5709492
	LRRK2-PD compared to IPD	2004	-3.1868641	0.81704537	-3.9004739	0.000100142	-4.7895049	-1.5842234
**AMR**	*GBA1*-PD compared to IPD	2130	-6.384889	2.36957582	-2.6945282	0.007270782	-11.039722	-1.7300555
	LRRK2-PD compared to IPD	2130	1.56649549	3.35396545	0.46705773	0.640649085	-5.0220891	8.15508008
**CAH**	*GBA1*-PD compared to IPD	805	-2.2487685	1.61532256	-1.3921483	0.164456992	-5.4219345	0.92439757
	LRRK2-PD compared to IPD	805	-0.4800511	3.35090351	-0.1432602	0.886138739	-7.0626207	6.1025186
**CAS**	*GBA1*-PD compared to IPD	750	-8.3624303	2.52479596	-3.3121212	0.000990552	-13.322529	-3.4023318
	LRRK2-PD compared to IPD	750	-3.3782603	4.77464406	-0.7075418	0.479548039	-12.758307	6.00178669
**EAS**	*GBA1*-PD compared to IPD	3338	-3.9501264	1.24562922	-3.1711896	0.001536811	-6.3927142	-1.5075387
	LRRK2-PD compared to IPD	3338	0.49235249	0.7343653	0.67044629	0.502636141	-0.9476841	1.93238912
**EUR**	*GBA1*-PD compared to IPD	29153	-2.0059495	0.29833892	-6.723727	1.82E-11	-2.5907188	-1.4211801
	LRRK2-PD compared to IPD	29153	-1.6194725	0.68378714	-2.3683869	0.017875402	-2.9597527	-0.2791923
**FIN**	*GBA1*-PD compared to IPD	121	-9.8602247	3.34081738	-2.9514408	0.004584882	-16.550096	-3.1703533
**MDE**	*GBA1*-PD compared to IPD	600	-5.469375	3.32618	-1.6443413	0.100780131	-12.005544	1.06679396
	LRRK2-PD compared to IPD	600	-4.4576453	1.87651765	-2.3754881	0.017929321	-8.1451296	-0.7701611
**SAS**	*GBA1*-PD compared to IPD	500	-7.6754414	2.49968794	-3.0705599	0.002281299	-12.589498	-2.7613851
	LRRK2-PD compared to IPD	500	5.37802819	13.0163133	0.413176	0.679697427	-20.210325	30.966381

Columns indicate the number of individuals included (N), regression coefficient (Coef.) representing the estimated AAO difference compared to IPD, standard error (Std.Err.), t-statistic (t), p-value (P), and 95% confidence interval ([0.025, 0.975]).

Supplementary Table 3. Summary of genetic findings across all ancestries. Columns represent genes investigated; rows show the number of variant carriers per study type within each ancestry group.

		PD risk	Typical autosomal-dominant PD					Early-onset recessive PD			Atypical parkinsonism					Dual carrier	Total (%)
		GBA1	*LRRK2*	*LRRK2 risk**	*SNCA*	*VPS35*	*RAB32*	*PINK1*	*PRKN*	*PARK7/DJ-1*	*ATP13A2*	*DCTN1*	*SLC20A2*	*RAB39B*	*WDR45*		
**AAC**	**Total affected**	**133**	**0**	**0**	**0**	**0**	**0**	**0**	**1**	**0**	**0**	**0**	**0**	**0**	**0**	**0**	**134/373 (35.9)**
	PD unselected cohort	121	0	0	0	0	0	0	0	0	0	0	0	0	0	0	121/352 (34.4)
	Monogenic recruitment	6	0	0	0	0	0	0	1	0	0	0	0	0	0	0	7/7 (100)
	Other phenotypes	4	0	0	0	0	0	0	0	0	0	0	0	0	0	0	4/12 (33.3)
	Genetically enriched affected	2	0	0	0	0	0	0	0	0	0	0	0	0	0	0	2/2 (100)
	**Total unaffected**	**222**	**2**	**1**	**0**	**0**	**0**	**0**	**0**	**0**	**0**	**0**	**0**	**0**	**0**	**0**	**225/855 (26.3)**
	Controls	218	1	1	0	0	0	0	0	0	0	0	0	0	0	0	220/838 (26.3)
	Healthy family members	0	1	0	0	0	0	0	0	0	0	0	0	0	0	0	1/1 (100)
	Population cohort	4	0	0	0	0	0	0	0	0	0	0	0	0	0	0	4/16 (25.0)
	Genetically enriched unaffected	0	0	0	0	0	0	0	0	0	0	0	0	0	0	0	0/0 (0)
**AFR**	**Total affected**	**532**	**1**	**2**	**0**	**0**	**0**	**0**	**1**	**0**	**0**	**0**	**0**	**0**	**0**	**0**	**536/1030 (52.0)**
	PD unselected cohort	522	1	2	0	0	0	0	1	0	0	0	0	0	0	0	526/1004 (52.4)
	Monogenic recruitment	6	0	0	0	0	0	0	0	0	0	0	0	0	0	0	6/13 (46.2)
	Other phenotypes	4	0	0	0	0	0	0	0	0	0	0	0	0	0	0	4/13 (30.8)
	Genetically enriched affected	0	0	0	0	0	0	0	0	0	0	0	0	0	0	0	0/0 (0)
	**Total unaffected**	**573**	**1**	**2**	**0**	**0**	**0**	**0**	**0**	**0**	**0**	**0**	**0**	**0**	**0**	**0**	**576/1743 (33.0)**
	Controls	570	1	2	0	0	0	0	0	0	0	0	0	0	0	0	573/1730 (33.1)
	Healthy family members	0	0	0	0	0	0	0	0	0	0	0	0	0	0	0	0/1 (0)
	Population cohort	3	0	0	0	0	0	0	0	0	0	0	0	0	0	0	3/12 (25.0)
	Genetically enriched unaffected	0	0	0	0	0	0	0	0	0	0	0	0	0	0	0	0/0 (0)
**AJ**	**Total affected**	**356**	**384**	**0**	**0**	**0**	**0**	**0**	**0**	**0**	**0**	**0**	**0**	**0**	**0**	**41**	**781/2045 (38.2)**
	PD unselected cohort	242	192	0	0	0	0	0	0	0	0	0	0	0	0	22	456/1632 (27.9)
	Monogenic recruitment	2	7	0	0	0	0	0	0	0	0	0	0	0	0	2	11/26 (42.3)
	Other phenotypes	12	2	0	0	0	0	0	0	0	0	0	0	0	0	1	15/87 (17.2)
	Genetically enriched affected	100	183	0	0	0	0	0	0	0	0	0	0	0	0	16	299/300 (99.7)
	**Total unaffected**	**258**	**232**	**0**	**0**	**0**	**0**	**0**	**0**	**0**	**0**	**0**	**0**	**0**	**0**	**26**	**516/1158 (44.6)**
	Controls	26	7	0	0	0	0	0	0	0	0	0	0	0	0	1	34/451 (7.5)
	Healthy family members	0	2	0	0	0	0	0	0	0	0	0	0	0	0	0	2/2 (100)
	Population cohort	18	6	0	0	0	0	0	0	0	0	0	0	0	0	0	24/242 (9.9)
	Genetically enriched unaffected	214	217	0	0	0	0	0	0	0	0	0	0	0	0	25	456/463 (98.5)

**AMR**	**Total affected**	**75**	**46**	**0**	**0**	**0**	**0**	**0**	**10**	**0**	**2**	**0**	**0**	**0**	**0**	**1**	**134/2143 (6.3)**
	PD unselected cohort	67	40	0	0	0	0	0	10	0	0	0	0	0	0	1	118/2019 (5.8)
	Monogenic recruitment	8	4	0	0	0	0	0	0	0	2	0	0	0	0	0	14/96 (14.6)
	Other phenotypes	0	0	0	0	0	0	0	0	0	0	0	0	0	0	0	0/25 (0)
	Genetically enriched affected	0	2	0	0	0	0	0	0	0	0	0	0	0	0	0	2/3 (66.7)
	**Total unaffected**	**14**	**3**	**0**	**0**	**0**	**0**	**0**	**0**	**0**	**0**	**0**	**0**	**0**	**0**	**0**	**17/1485 (1.1)**
	Controls	12	1	0	0	0	0	0	0	0	0	0	0	0	0	0	13/1459 (0.9)
	Healthy family members	0	0	0	0	0	0	0	0	0	0	0	0	0	0	0	0/2 (0)
	Population cohort	0	0	0	0	0	0	0	0	0	0	0	0	0	0	0	0/20 (0)
	Genetically enriched unaffected	2	2	0	0	0	0	0	0	0	0	0	0	0	0	0	4/4 (100)
**CAH**	**Total affected**	**127**	**20**	**4**	**1**	**2**	**0**	**0**	**7**	**0**	**0**	**0**	**0**	**0**	**0**	**0**	**161/813 (19.8)**
	PD unselected cohort	122	15	4	1	2	0	0	7	0	0	0	0	0	0	0	151/785 (19.2)
	Monogenic recruitment	2	4	0	0	0	0	0	0	0	0	0	0	0	0	0	6/11 (54.5)
	Other phenotypes	3	0	0	0	0	0	0	0	0	0	0	0	0	0	0	3/17 (17.6)
	Genetically enriched affected	0	1	0	0	0	0	0	0	0	0	0	0	0	0	0	1/1 (100)
	**Total unaffected**	**37**	**5**	**1**	**0**	**0**	**0**	**0**	**0**	**0**	**0**	**0**	**0**	**0**	**0**	**0**	**43/339 (12.7)**
	Controls	36	2	1	0	0	0	0	0	0	0	0	0	0	0	0	39/323 (12.1)
	Healthy family members	0	3	0	0	0	0	0	0	0	0	0	0	0	0	0	3/4 (75.0)
	Population cohort	0	0	0	0	0	0	0	0	0	0	0	0	0	0	0	0/10 (0)
	Genetically enriched unaffected	1	0	0	0	0	0	0	0	0	0	0	0	0	0	0	1/2 (50.0)
**CAS**	**Total affected**	**30**	**2**	**11**	**0**	**0**	**0**	**0**	**4**	**0**	**0**	**0**	**0**	**0**	**0**	**1**	**48/756 (6.3)**
	PD unselected cohort	24	1	9	0	0	0	0	4	0	0	0	0	0	0	0	38/625 (6.1)
	Monogenic recruitment	6	0	2	0	0	0	0	0	0	0	0	0	0	0	1	9/107 (8.4)
	Other phenotypes	0	0	0	0	0	0	0	0	0	0	0	0	0	0	0	0/21 (0)
	Genetically enriched affected	0	1	0	0	0	0	0	0	0	0	0	0	0	0	0	1/2 (50.0)
	**Total unaffected**	**9**	**1**	**3**	**0**	**0**	**0**	**0**	**0**	**0**	**0**	**0**	**0**	**0**	**0**	**0**	**13/371 (3.5)**
	Controls	9	0	3	0	0	0	0	0	0	0	0	0	0	0	0	12/348 (3.4)
	Healthy family members	0	0	0	0	0	0	0	0	0	0	0	0	0	0	0	0/21 (0)
	Population cohort	0	0	0	0	0	0	0	0	0	0	0	0	0	0	0	0/1 (0)
	Genetically enriched unaffected	0	1	0	0	0	0	0	0	0	0	0	0	0	0	0	1/1 (100)
**EAS**	**Total affected**	**116**	**12**	**441**	**4**	**3**	**0**	**14**	**14**	**0**	**0**	**1**	**2**	**0**	**0**	**17**	**624/3394 (18.4)**
	PD unselected cohort	24	8	215	1	2	0	4	2	0	0	0	0	0	0	2	258/1894 (13.6)
	Monogenic recruitment	89	2	216	3	1	0	5	12	0	0	1	2	0	0	15	346/1412 (24.5)
	Other phenotypes	1	0	8	0	0	0	0	0	0	0	0	0	0	0	0	9/57 (15.8)
	Genetically enriched affected	2	2	2	0	0	0	5	0	0	0	0	0	0	0	0	11/31 (35.5)

	**Total unaffected**	**19**	**1**	**217**	**0**	**0**	**0**	**0**	**0**	**0**	**0**	**0**	**0**	**0**	**0**	**1**	**238/2503 (9.5)**
	Controls	18	1	208	0	0	0	0	0	0	0	0	0	0	0	1	228/2468 (9.2)
	Healthy family members	1	0	9	0	0	0	0	0	0	0	0	0	0	0	0	10/33 (30.3)
	Population cohort	0	0	0	0	0	0	0	0	0	0	0	0	0	0	0	0/2 (0)
	Genetically enriched unaffected	0	0	0	0	0	0	0	0	0	0	0	0	0	0	0	0/0 (0)
**EUR**	**Total affected**	**2610**	**435**	**31**	**60**	**4**	**7**	**8**	**65**	**1**	**0**	**4**	**0**	**2**	**1**	**34**	**3262/29339 (11.1)**
	PD unselected cohort	2165	226	25	14	4	5	7	54	1	0	4	0	2	1	22	2530/24180 (10.5)
	Monogenic recruitment	148	18	0	15	0	2	1	8	0	0	0	0	0	0	4	196/1465 (13.4)
	Other phenotypes	188	4	6	0	0	0	0	1	0	0	0	0	0	0	0	199/3214 (6.2)
	Genetically enriched affected	109	187	0	31	0	0	0	2	0	0	0	0	0	0	8	337/480 (70.2)
	**Total unaffected**	**998**	**189**	**19**	**15**	**0**	**0**	**0**	**1**	**0**	**0**	**0**	**0**	**0**	**0**	**11**	**1233/19795 (6.2)**
	Controls	505	21	8	0	0	0	0	0	0	0	0	0	0	0	5	539/10518 (5.1)
	Healthy family members	12	1	0	2	0	0	0	0	0	0	0	0	0	0	0	15/95 (15.8)
	Population cohort	419	12	11	1	0	0	0	1	0	0	0	0	0	0	0	444/8926 (5.0)
	Genetically enriched unaffected	62	155	0	12	0	0	0	0	0	0	0	0	0	0	6	235/256 (91.8)
**FIN**	**Total affected**	**14**	**0**	**0**	**0**	**0**	**0**	**0**	**0**	**0**	**0**	**0**	**0**	**0**	**0**	**0**	**14/121 (11.6)**
	PD unselected cohort	13	0	0	0	0	0	0	0	0	0	0	0	0	0	0	13/111 (11.7)
	Monogenic recruitment	0	0	0	0	0	0	0	0	0	0	0	0	0	0	0	0/0 (0)
	Other phenotypes	1	0	0	0	0	0	0	0	0	0	0	0	0	0	0	1/9 (11.1)
	Genetically enriched affected	0	0	0	0	0	0	0	0	0	0	0	0	0	0	0	0/1 (0)
	**Total unaffected**	**1**	**0**	**0**	**0**	**0**	**0**	**0**	**0**	**0**	**0**	**0**	**0**	**0**	**0**	**0**	**1/14 (7.1)**
	Controls	0	0	0	0	0	0	0	0	0	0	0	0	0	0	0	0/9 (0)
	Healthy family members	0	0	0	0	0	0	0	0	0	0	0	0	0	0	0	0/1 (0)
	Population cohort	1	0	0	0	0	0	0	0	0	0	0	0	0	0	0	1/4 (25.0)
	Genetically enriched unaffected	0	0	0	0	0	0	0	0	0	0	0	0	0	0	0	0/0 (0)
**MDE**	**Total affected**	**19**	**65**	**0**	**1**	**0**	**1**	**10**	**4**	**0**	**0**	**0**	**0**	**0**	**0**	**4**	**104/620 (16.8)**
	PD unselected cohort	13	41	0	0	0	1	10	4	0	0	0	0	0	0	3	72/484 (14.9)
	Monogenic recruitment	5	1	0	1	0	0	0	0	0	0	0	0	0	0	0	7/89 (7.8)
	Other phenotypes	1	0	0	0	0	0	0	0	0	0	0	0	0	0	0	1/23 (4.3)
	Genetically enriched affected	0	23	0	0	0	0	0	0	0	0	0	0	0	0	1	24/24 (100)
	**Total unaffected**	**0**	**3**	**0**	**0**	**0**	**0**	**0**	**0**	**0**	**0**	**0**	**0**	**0**	**0**	**0**	**3/244 (1.2)**
	Controls	0	0	0	0	0	0	0	0	0	0	0	0	0	0	0	0/224 (0)
	Healthy family members	0	0	0	0	0	0	0	0	0	0	0	0	0	0	0	0/6 (0)
	Population cohort	0	1	0	0	0	0	0	0	0	0	0	0	0	0	0	1/12 (8.3)
	Genetically enriched unaffected	0	2	0	0	0	0	0	0	0	0	0	0	0	0	0	2/2 (100)

**SAS**	**Total affected**	**31**	**1**	**1**	**0**	**0**	**0**	**0**	**5**	**0**	**0**	**0**	**0**	**0**	**0**	**0**	**38/505 (7.5)**
	PD unselected cohort	8	0	0	0	0	0	0	2	0	0	0	0	0	0	0	10/243 (4.1)
	Monogenic recruitment	20	1	1	0	0	0	0	1	0	0	0	0	0	0	0	23/195 (11.8)
	Other phenotypes	1	0	0	0	0	0	0	2	0	0	0	0	0	0	0	3/61 (4.9)
	Genetically enriched affected	2	0	0	0	0	0	0	0	0	0	0	0	0	0	0	2/6 (33.3)
	**Total unaffected**	**3**	**0**	**0**	**0**	**0**	**0**	**0**	**0**	**0**	**0**	**0**	**0**	**0**	**0**	**0**	**3/235 (1.3)**
	Controls	0	0	0	0	0	0	0	0	0	0	0	0	0	0	0	0/217 (0)
	Healthy family members	3	0	0	0	0	0	0	0	0	0	0	0	0	0	0	3/18 (16.7)
	Population cohort	0	0	0	0	0	0	0	0	0	0	0	0	0	0	0	0/0 (0)
	Genetically enriched unaffected	0	0	0	0	0	0	0	0	0	0	0	0	0	0	0	0/0 (0)

* *LRRK2* PD risk variants include rs33949390 (chr12:40320043:G:C, p.R1628P) and rs34778348 (chr12:40363526:G:A, p.G2385R).
** Dual carriers refer to carriers of two pathogenic/likely pathogenic or PD risk variants in two different genes.
AAC = African admixed, AFR = African, AJ = Ashkenazi Jewish, AMR = Latinos and Indigenous people of the Americas, CAH = Complex admixture, CAS = Central Asian, EAS = East Asian, EUR = European, FIN = Finnish, MDE = Middle East, SAS = South Asian.

Supplementary Table 4. List of all identified variants and concordance check NBA and CES/WGS.

Variant	AA change	carrier total*	NBA available	carrier NBA	CES available	carrier CES	WGS available	carrier WGS	NBA + CES available	Valid. (%)	NBA missed	NBA + WGS available	Valid. (%)	NBA missed	comment	cluster plot	IN or EX?	comment on inclusion/exclusion
GBA1																		
chr1:155235002:C:T	R535H	43	43	43	9	0	8	6	9			8	6/8 (75)		only WGS Gauchian detected	yes	IN	validation with CES/WGS
chr1:155235195:C:T	R502H	2	2	2	0	0	0	0	0			0			no matching CES/WGS data	yes	IN	
chr1:155235196:G:A	R502C	96	93	93	11	11	19	19	9	10/10 (100)		17	17/17 (100)			yes	IN	validation with CES/WGS
chr1:155235205:C:G	V499L	1	0	0	1	0	1	1	0			0			only WGS Gauchian detected	yes	IN	
chr1:155235205:C:T	V499M	12	12	12	2	0	1	1	0	0/2 (0)		1	1/1 (100)			yes	IN	validation with CES/WGS
chr1:155235241:C:T	A487T	3	3	0	0	0	3	3	0			3			only WGS detected	no	IN	only CES/WGS detected
chr1:155235252:A:C	L483R	15	15	0	0	0	15	15	0			15			only WGS Gauchian detected	no	IN	only CES/WGS detected
chr1:155235252:A:G	L483P	85	81	0	0	0	85	85	0			75			only WGS Gauchian detected	no	IN	only CES/WGS detected
chr1:155235256:C:T	D482N	1	1	1	0	0	0	0	0			0				yes	IN	
chr1:155235680:C:T	c.1388+1G>A	1	1	0	0	0	1	1	0			1			only WGS detected	no	IN	only CES/WGS detected
chr1:155235716:G:T	Y451*	1	1	0	1	1	0	0	1			0			only CES detected	no	IN	only CES/WGS detected
chr1:155235727:C:G	D448H	19	15	0	6	6	13	13	5			10			only CES/WGS detected	no	IN	only CES/WGS detected
chr1:155235772:C:A	V433L	9	8	8	4	4	1	0	2	3/3 (100)		1	0/1 (0)			yes	IN	validation with CES/WGS
chr1:155235773:C:T	W432*	2	2	2	0	0	1	1	0			1	1/1 (100)			yes	IN	validation with CES/WGS
chr1:155235777:T:C	N431S	3	3	3	0	0	0	0	0			0			no matching CES/WGS data	yes	EX	
chr1:155235780:G:T	P430H	1	1	0	0	0	1	1	0			1			only WGS detected	no	IN	only CES/WGS detected
chr1:155235798:A:G	L424P	5	5	5	1	0	2	2	1	1/1 (100)		2	2/2 (100)			yes	IN	validation with CES/WGS
chr1:155235804:AG:A	Ala423Profs*2	21	20	0	0	0	21	21	0			20			only WGS Gauchian	yes	IN	only CES/WGS detected

															detected			
chr1:155235810:C:T	W420*	2	2	2	0	0	0	0	0			0			no matching CES/WGS data	yes	EX	
chr1:155235819:C:T	W417*	2	2	2	0	0	0	0	0			0			no matching CES/WGS data	yes	EX	
chr1:155235823:C:T	G416S	27	27	27	4	3	2	1	4	3/4 (75)		2	1/2 (50)			yes	IN	validation with CES/WGS
chr1:155235829:C:A	V414L	1	0	0	1	1	0	0	0			0			only CES detected	no	IN	only CES/WGS detected
chr1:155235829:C:G	V414L	1	1	0	0	0	1	1	0			1			only WGS detected	no	IN	only CES/WGS detected
chr1:155235843:T:C	N409S	1084	1034	1033	99	99	469	469	80	80/80 (100)		438	436/438 (100)			yes	IN	validation with CES/WGS; cluster plot
chr1:155236246:G:A	T408M	1376	1336	1336	127146	146	136	135	120	120/12 0 (100)		115	114/114 100)			yes	IN	validation with CES/WGS; cluster plot
chr1:155236277:G:A	R398*	2	2	0	1	1	1	1	1			1			only CES or WGS detected	no	IN	only CES/WGS detected
chr1:155236295:G:A	R392W	1	1	0	1	1	0	0	1			0			only CES detected	no	IN	only CES/WGS detected
chr1:155236367:G:A	R368C	5	5	5	0	0	0	0	0			0			no matching CES/WGS data	yes	EX	
chr1:155236375:T:TC	E365Gfs*71	1	1	0	1	1	0	0	1			0			only CES detected	no	IN	only CES/WGS detected
chr1:155236376:C:T	E365K	2004	1937	1923	250	250	238	238	205	197/19 7 (100)	8/205	219	213/213 (100)	6/219		yes	IN	validation with CES/WGS; cluster plot
chr1:155236381:A:G	L363P	4	4	4	0	0	3	2	0			3	2/3 (67)			yes	IN	validation with CES/WGS
chr1:155236384:G:A	T362I	10	10	9	2	2	1	1	2	2/2 (100)		1		1/1		yes	IN	validation with CES/WGS
chr1:155236417:C:G	W351S	1	1	1	0	0	1	1	0			1	1/1 (100)			no	IN	validation with CES/WGS
chr1:155236439:CA:C	Y343*	1	1	0	0	0	1	1	0			1			only WGS detected	no	IN	only CES/WGS detected
chr1:155237412:T:C	S310G	6	6	4	0	0	2	2	0			2				no	IN	only CES/WGS detected
chr1:155237425:AG:A	P305Lfs*31	6	6	0	1	1	5	5	1		1/1	5		5/5	only CES or WGS detected	no	IN	only CES/WGS detected
chr1:155237441:GC:G	A300Pfs*4	1	1	0	0	0	1	1	0			1			only WGS detected	no	IN	only CES/WGS detected
chr1:155237444:A:G	I299T	2	1	0	1	1	1	1	0			1			only CES or WGS detected	no	IN	only CES/WGS detected
chr1:155237453:C:T	R296Q	35	35	35	7	6	6	5	7	6/7 (86)		6	5/6 (83)			yes	IN	validation with CES/WGS
chr1:155237576:A:T	F255Y	5	4	0	4	4	1	1	3		3/3	1		1/1	only CES or WGS	no	IN	only CES/WGS detected

															detected			
chr1:155237579:C:G	c.762-1G>C	1	1	0	0	0	1	1	0			1		1/1	only WGS detected	no	IN	only CES/WGS detected
chr1:155237580:T:C	c.762-2A>G	1	1	0	0	0	1	1	0			1		1/1	only WGS detected	no	IN	only CES/WGS detected
chr1:155238141:A:T	F252I	7	7	0	3	3	4	4	3		3/3	4		4/4	only CES or WGS detected	no	IN	only CES/WGS detected
chr1:155238174:C:T	G241R	12	12	0	5	4	4	4	5		5/5	4		4/4	only CES or WGS detected	yes	IN	only CES/WGS detected
chr1:155238192:A:G	S235P	4	3	0	2	2	2	2	2		2/2	1		1/1	only CES or WGS detected	no	IN	only CES/WGS detected
chr1:155238194:C:T	G234E	1	1	0	1	1	0	0	1		1/1	0			only CES detected	no	IN	only CES/WGS detected
chr1:155238206:A:C	V230G	3	3	3	0	0	1	1	0			1	1/1 (100)			yes	IN	validation with CES/WGS
chr1:155238214:A:C	N227K	7	7	7	0	0	3	3	0			3	3/3 (100)			yes	IN	validation with CES/WGS
chr1:155238215:T:C	N227S	18	18	17	2	2	1	1	2	1/1 (100)	1/2	1	1/1 (100)			yes	IN	validation with CES/WGS
chr1:155238228:A:G	W223R	11	11	10	0	0	2	2	0			2	1/1 (100)	1/2		yes	EX	
chr1:155238234:G:T	V230G	1	1	1	0	0	0	0	0			0				yes	EX	
chr1:155238242:C:T	W218*	2	2	2	0	0	1	1	0			1	1/1 (100)			yes	IN	validation with CES/WGS
chr1:155238260:G:C	S212*	1	1	0	0	0	1	1	0			1		1/1	only WGS detected	no	IN	only CES/WGS detected
chr1:155238264:CG:C	V211Ffs*20	3	3	0	0	0	3	3	0			3		3/3	only WGS detected	no	IN	only CES/WGS detected
chr1:155238291:G:A	R202*	2	2	0	2	2	0	0	2			0			only CES detected	no	IN	only CES/WGS detected
chr1:155238298:CAG:C	L199Dfs*62	2	1	0	0	0	2	2	0			1			only WGS detected	no	IN	only CES/WGS detected
chr1:155238307:C:T	c.589-1G>A	2	2	2	0	0	1	0	0			1	0/1 (0)			yes	EX	
chr1:155238519:T:G	K196Q	4	3	0	1	1	3	3	1			2			only CES or WGS detected	no	IN	only CES/WGS detected
chr1:155238571:AG:A	P178Lfs*22	1	1	0	0	0	1	1	0			1			only WGS detected	no	IN	only CES/WGS detected
chr1:155238596:C:A	R170L	2	2	0	0	0	2	2	0			2			only WGS detected	no	IN	only CES/WGS detected
chr1:155238597:G:A	R170C	4	4	0	0	0	4	4	0			4		4/4	only WGS detected	no	IN	only CES/WGS detected
chr1:155238617:GC:G	A163Pfs*37	2	1	0	0	0	2	2	0			1			only WGS detected	no	IN	only CES/WGS detected
chr1:155238629:C:T	R159Q	7	7	7	0	0	1	1	0			1	1/1 (100)			yes	IN	validation with CES/WGS
chr1:155238630:G:A	R159W	4	4	0	0	0	4	4	0			4		4/4	only WGS detected	no	IN	only CES/WGS detected

chr1:155239633:G:A	S146L	5	5	5	1	1	0	0	1	1/1 (100)		0				yes	IN	validation with CES/WGS
chr1:155239933:C:T	R87Q	1	1	0	0	0	1	1	0			1		1/1	only WGS detected	no	IN	only CES/WGS detected
chr1:155239934:G:A	R87W	4	4	4	1	1	1	0	1	1/1 (100)		1	0/1 (0)			yes	IN	validation with CES/WGS
chr1:155239937:G:A	R86*	3	3	3	0	0	0	0	0			0			no matching CES/WGS data	no	EX	
chr1:155239939:C:T	G85E	1	1	1	0	0	1	1	0			1	1/1 (100)			no	IN	validation with CES/WGS
chr1:155239968:GGTA:G	T75del	1	1	0	0	0	1	1	0			1		1/1	only WGS detected	no	IN	only CES/WGS detected
chr1:155239989:CG:C	P68Rfs*23	1	1	0	0	0	1	1	0			1			only WGS detected	no	IN	only CES/WGS detected
chr1:155240025:G:GAC	C57Sfs*35	1	0	0	1	1	0	0	0			0			only CES detected	no	IN	only CES/WGS detected
chr1:155240629:C:T	c.115+1G>A	22	22	0	8	8	14	14	8		8/8	14		14/14	only CES and WGS detected	no	IN	only CES/WGS detected
chr1:155240660:G:GC	L29Afs*18	21	16	0	10	10	11	11	8		8/8	8		8/8	only CES and WGS detected	no	IN	only CES/WGS detected
RecNciI		24	22	0	0	0	24	24	0			22			only WGS Gauchian detected	no	IN	only CES/WGS detected
LRRK2																		
chr12:40298346:G:A	R1067Q	6	6	5	0	0	1	1	0			1	1/1 (100)			yes	IN	validation with CES/WGS
chr12:40308481:G:A	R1325Q	43	42	42	2	2	3	3	2	2/2 (100)		2	2/2 (200)			yes	IN	validation with CES/WGS
chr12:40310434:C:G	R1441G	102	95	95	0	0	36	36	0			29	29/29 (100)			yes	IN	validation with CES/WGS
chr12:40310434:C:T	R1441C	62	61	61	10	10	8	8	10	10/10 (100)		7	6/6 (100)	1/7		yes	IN	validation with CES/WGS
chr12:40310435:G:A	R1441H	14	14	13	1	1	1	1	1	1/1 (100)		1	1/1 (100)			yes	IN	validation with CES/WGS
chr12:40320043:G:C	R1628P	389	386	384	11	10	102	102	10	9/10 (90)		99	98/99 (99)			yes	IN	validation with CES/WGS; cluster plot
chr12:40321114:A:G	Y1699C	5	5	5	1	0	1	1	1	0/1 (0)		1	1/1 (100)			yes	IN	validation with CES/WGS
chr12:40322386:G:T	L1795F	10	10	10	0	0	4	4	0			4	4/4 (100)			yes	IN	validation with CES/WGS
chr12:40340400:G:A	G2019S	1295	1209	1206	147	146	588	586	121	119/12	1/121	528	527/528			yes	IN	validation with

										0 (99)			(100)					CES/WGS; cluster plot
chr12:40340404:T:C	I2020T	12	12	12	0	0	2	1	0			2	1/2 (50)			yes	IN	
chr12:40363526:G:A	G2385R	382	381	380	7	7	99	99	6	5/5 (100)	1/6	117	117/117 (100)			yes	IN	validation with CES/WGS; cluster plot
SNCA																		
chr4:89828149:C:T	A53T	59	51	51	0	0	38	38	0			30	30/30 (100)			yes	IN	validation with CES/WGS
chr4:89828154:C:T	G51D	3	3	3	0	0	1	1	0			1	1/1 (100)			yes	IN	validation with CES/WGS
VPS35																		
chr16:46662452:C:T	D620N	10	9	8	4	4	1	1	3	3/3 (100)		1	1/1 (100)			no	IN	validation with CES/WGS
RAB32																		
chr6:146544084:C:G	S71R	9	8	0	0	0	9	9	0			9			only wgs detected	no	IN	only CES/WGS detected
PINK1																		
chr1:20633808:TGCGGGCCTGGGGCTGC:T	W90Lfs*12	1	0	0	0	0	1	1	0			0			only WGS detected	no	IN	only CES/WGS detected
chr1:20633933:C:T	Q129*	2	2	2	0	0	0	0	0			0			no matching CES/WGS data	yes	EX	
chr1:20637963:T:G	V170G	1	1	1	0	0	0	0	0			0			no matching CES/WGS data	yes	EX	
chr1:20638119:G:A	W222*	1	1	0	0	0	1	1	0			1			only WGS detected	yes	IN	only CES/WGS detected
chr1:20639952:C:T*	R246*	13	13	13	3	0	1	0	3	0/3 (0)		1	0/1 (0)			no	EX	
chr1:20644639:G:A	G309D	1	1	1	0	0	0	0	0			0			no matching CES/WGS data	yes	EX	
chr1:20644639:G:A*	G309D	1	1	1	0	0	0	0	0			0			no matching CES/WGS data	yes	EX	
chr1:20644651:C:T*	T313M	126	126	126	3	0	9	0	3	0/3 (0)		9	0/9 (0)			no	EX	
chr1:20645640:T:C	L347P	19	18	17	0	0	8	8	0			7	6/6 (100)	1/7		yes	IN	validation with CES/WGS
chr1:20645640:T:C*	L347P	9	9	9	0	0	1	1	0			1	1/1 (100)			yes	IN	validation with CES/WGS
chr1:20648534:T:G*	F385V	1	1	1	0	0	0	0	0			0			no matching CES/WGS data	no	EX	
chr1:20649070:GC:G*	Y444Mfs*39	2	1	0	1	1	1	1	1		1/1	0		1/1	only CES or WGS detected	no	IN	only CES/WGS detected

chr1:20649109:C:T	Q456*	15	15	15	0	0	5	5	0			5	5/5 (100)			yes	IN	validation with CES/WGS
chr1:20649109:C:T*	Q456*	20	19	19	0	0	1	1	0			0				yes	IN	validation with CES/WGS
chr1:20649217:C:T*	R492*	26	26	26	4	0	4	0	4	0/4 (0)		4	0/4 (0)			no	EX	
chr1:20650506:AAT:A*	N521Tfs*40	1	1	0	0	0	1	1	0			1		1/1	only WGS detected	no	IN	only CES/WGS detected
chr1:20650541:C:CCAA*	Q534dup	1	0	0	0	0	1	1	0			0			only WGS detected	no	IN	only CES/WGS detected
PRKN																		
chr6:161350125:T:G*	M458L	1	1	0	0	0	1	1	0			1			only WGS detected	no	IN	only CES/WGS detected
chr6:161350144:GC:G*	C451Sfs*190	2	2	0	2	2	0	0	2		2/2	0			only CES detected	no	IN	only CES/WGS detected
chr6:161350163:C:T*	W445X	4	4	4	2	1	0	0	2	1/2 (50)		0				no	IN	validation with CES/WGS
chr6:161350176:A:G*	C441R	17	17	14	1	0	5	5	1	0/1 (0)		5	2/2 (100)	3/5		no	IN	validation with CES/WGS
chr6:161350208:C:T*	G430D	55	53	53	10	10	11	11	8	8/8 (100)		11	11/11 (100)			no	IN	validation with CES/WGS
chr6:161360121:A:G	C418R	1	1	0	0	0	1	1	0			1		1/1	only WGS detected	no	IN	only CES/WGS detected
chr6:161360129:G:T	T415N	1	1	1	0	0	0	0	0			0			no matching CES/WGS data	yes	IN	only CES/WGS detected
chr6:161548853:C:A*	c.1083+1G>T	2	2	0	0	0	2	2	0			2			only WGS detected	no	IN	only CES/WGS detected
chr6:161548853:C:T	c.1083+1G>A	1	1	1	0	0	0	0	0			0			no matching CES/WGS data	yes	IN	
chr6:161569403:GTT:G*	N295Lfs*4	1	1	0	0	0	1	1	0			1		1/1	only WGS detected	no	IN	only CES/WGS detected
chr6:161785793:C:G*	G284R	3	3	0	0	0	3	3	0			3		3/3	only WGS detected	no	IN	only CES/WGS detected
chr6:161785820:G:A*	R275W	161	152	100	35	35	15	15	33	12/12 (100)	21/33	45	14/14 (100)	31/45		yes	IN	validation with CES/WGS
chr6:161785820:G:A	R275W	9	9	9	0	0	1	1	0			1	1/1 (100)			yes	IN	validation with CES/WGS
chr6:161785856:A:C	C263G	2	2	0	0	0	2	2	0			2		2/2	only WGS detected	no	IN	only CES/WGS detected
chr6:161785885:C:A	C253F	1	1	1	0	0	0	0	0			0			no matching CES/WGS data	yes	IN	
chr6:161785885:C:T*	C253Y	3	3	0	0	0	3	3	0			3		3/3	only WGS detected	no	IN	only CES/WGS detected
chr6:161973317:G:A*	T240M	37	37	27	2	0	4	2	2	0/2 (0)		4	1/3 (33)	1/4		yes	IN	
chr6:161973317:G:A	T240M	1	1	1	0	0	0	0	0			0			no matching	yes	IN	

															CES/WGS data			
chr6:161973322:G:C	C238W	1	1	1	0	0	0	0	0			0			no matching CES/WGS data	yes	IN	
chr6:161973403:T:A	K211N	2	2	0	1	1	1	1	1		1/1	1		1/1	only CES or WGS detected	no	IN	only CES/WGS detected
chr6:161973418:C:T	c.619-1G>A	3	3	3	0	0	0	0	0			0			no matching CES/WGS data	yes	EX	
chr6:162201154:G:A*	Q171*	1	1	0	0	0	1	1	0			1		1/1	only WGS detected	no	IN	only CES/WGS detected
chr6:162201248:AC:A*	G139Vfs*38	1	1	0	1	1	0	0	1		1/1	0			only CES detected	no	IN	only CES/WGS detected
chr6:162262543:G:GT	P132Tfs*9	1	1	0	0	0	1	1	0			1		1/1	only WGS detected	no	IN	only CES/WGS detected
chr6:162262560:TCAGTGTGCAGAATGACAGCCAGCCCCACAGAGTCTCCTGG:T	P113Tfs*51	5	5	0	0	0	5	5	0			5		5/5	only WGS detected	no	IN	only CES/WGS detected
chr6:162262560:TCAGTGTGCAGAATGACAGCCAGCCCCACAGAGTCTCCTGG:T*	P113Tfs*51	28	26	0	11	11	17	17	11		11/11	15		15/15	only CES or WGS detected	no	IN	only CES/WGS detected
chr6:162262702:C:A*	E79*	5	4	4	1	1	1	0	1		1/1	1	0/1 (0)			no	EX	
chr6:162262715:C:CCA*	W74Cfs*8	2	2	0	0	0	2	2	0			2		2/2	only WGS detected	no	IN	only CES/WGS detected
chr6:162443309:C:T*	c.171+1G>A	1	2	0	1	1	0	0	1		1/1	0			only CES detected	no	IN	only CES/WGS detected
chr6:162443314:A:T*	V56E	1	1	0	1	1	0	0	1		1/1	0			only CES detected	no	IN	only CES/WGS detected
chr6:162443325:AT:A	N52Mfs*29	5	3	0	3	3	2	2	2		2/2	3		3/3	only CES and WGS detected	no	IN	only CES/WGS detected
chr6:162443325:AT:A*	N52Mfs*29	23	19	0	10	10	13	13	7		7/7	12		12/12	only CES and WGS detected	no	IN	only CES/WGS detected
chr6:162443356:C:G*	R42P	6	6	0	1	1	5	5	1		1/1	5		5/5	only CES and WGS detected	no	IN	only CES/WGS detected
chr6:162443365:TC:T*	D39Tfs*5	1	1	0	0	0	1	1	0			1		1/1	only WGS detected	no	IN	only CES/WGS detected
chr6:162443378:CCT:C	Q34Rfs*5	2	2	0	1	1	1	1	1		1/1	1		1/1	only CES or WGS detected	no	IN	only CES/WGS detected
chr6:162443378:CCT:C*	Q34Rfs*5	17	14	0	5	5	12	12	3		3/3	11		11/11	only CES or WGS detected	no	IN	only CES/WGS detected
chr6:162443379:CT:C	Q34Rfs*10	2	2	0	2	2	0	0	2		2/2	0			only CES detected	no	IN	only CES/WGS detected
chr6:162443383:C:T*	R33Q	2	2	1	1	1	0	0	1		1/1	0				no	IN	validation with CES/WGS

chr6:162443384:G:A	R33*	1	1	1	0	0	0	0	0			0			no matching CES/WGS data	yes	IN	
chr6:162443408:G:A	Q25*	1	1	0	1	1	0	0	1		1/1	0			only CES detected	no	IN	only CES/WGS detected
chr6:162727667:A:G*	M1?	28	28	28	3	0	3	0	3	0/3 (0)	3	0/3 (0)				yes	EX	
chr6:162727667:A:G	M1?	1	1	1	0	0	0	0	0			0			no matching CES/WGS data	yes	EX	
PARK7/DJ-1																		
chr1:7962868:G:A	R28Q	1	1	0	0	0	1	1	0			1		1/1	only WGS detected	no	IN	only CES/WGS detected
chr1:7965336:G:GT*	A36Cfs*12	2	2	0	2	2	0	0	2		2/2	0			only CES detected	no	IN	only CES/WGS detected
chr1:7965425:G:C	E64D	1	1	1	0	0	0	0	0			0			no matching CES/WGS data	no	IN	
chr1:7965425:G:C*	E64D	4	4	4	0	0	1	1	0			1	1/1 (100)			no	IN	validation with CES/WGS
ATP13A2																		
chr1:16986346:G:A*	Q1140*	14	14	14	2	0	0	0	2	0/2 (0)		0				no	EX	
chr1:16987071:AG:A*	Y1020Tfs*3	11	9	0	5	5	6	6	4		4/4	5		5/5	only CES or WGS detected	no	IN	only CES/WGS detected
chr1:16988455:C:T*	G877R	2	2	2	0	0	0	0	0			0			no matching CES/WGS data	no	EX	only CES/WGS detected
chr1:16989886:C:T*	c.2529+1G>A	1	1	0	0	0	1	1	0			1		1/1	only WGS detected	no	IN	only CES/WGS detected
chr1:16989961:G:A*	R819*	12	12	12	5	0	0	0	5	0/5 (0)		0				no	EX	
chr1:16992345:G:A	Q635*	1	1	0	1	1	0	0	1		1/1	0			only CES detected	no	IN	only CES/WGS detected
chr1:16996381:C:T*	1306+5G>A	1	1	0	0	0	1	1	0			1		1/1	only WGS detected	no	IN	only CES/WGS detected
chr1:17000466:C:T*	W258*	2	2	2	0	0	0	0	0			0			no matching CES/WGS data	no	EX	
chr1:17004690:A:C	c.477+2T>G	2	1	0	2	2	0	0	1		1/1	0			only CES detected	no	IN	only CES/WGS detected
chr1:17005376:CTT:C	L95Rfs*3	1	1	0	0	0	1	1	0			1		1/1	only WGS detected	no	IN	only CES/WGS detected
DCTN1																		
chr2:74363626:C:A	E1067*	3	3	0	0	0	3	3	0			3			only WGS detected	no	IN	only CES/WGS detected
chr2:74367699:A:T	Y727*	1	1	1	0	0	1	1	0			1	1/1 (100)			no	IN	validation with CES/WGS
chr2:74368787:GC:G	P599Qfs*23	1	1	0	1	1	0	0	1		1/1	0			only CES detected	no	IN	only CES/WGS detected
chr2:74377718:T:TCTGCTCCATCTTCAAATTGA	F97Qfs*45	1	0	0	1	1	0	0	0			0			only CES detected	no	IN	only CES/WGS detected

RAB39B																		
chrX:155260871:C:T	G192R	2	1	0	0	0	2	2	0			1			only WGS detected	no	IN	only CES/WGS detected
chrX:155260982:G:A*	R155*	1	1	0	1	1	0	0	1			0			only CES detected	no	IN	only CES/WGS detected
SLC20A2																		
chr8:42430121:C:T	W551*	3	3	3	0	0	2	0	0			2	0/2 (0)			yes	EX	
chr8:42437354:G:T	Y386*	1	1	0	0	0	1	1	0			1			only WGS detected	no	IN	only CES/WGS detected
chr8:42439624:G:A	R254*	3	3	3	0	0	0	0	0			0			no matching CES/WGS data	yes	EX	
chr8:42444645:C:T	c.730+1G>A	20	20	20	2	0	1	0	2	0/2 (0)		1	0/1 (0)			yes	EX	
chr8:42465875:G:A	P111L	1	1	1	0	0	1	1	0			1			only WGS detected	no	IN	only CES/WGS detected
WDR45																		
chrX:49074913:C:T	c.974-1C>T	55	55	55	2	0	2	0	2	0/2 (0)		2	0/2 (0)			yes	EX	
chrX:49075282:C:G	c.828-1G>C	1	1	1	0	0	0	0	0			0			no matching CES/WGS data	yes	EX	
chrX:49075573:G:A	R233*	1	1	1	0	0	0	0	0			0			no matching CES/WGS data	yes	EX	
chrX:49076469:G:A	R133*	1	1	0	1	1	0	0	1		1/1	0			only CES detected	no	IN	only CES/WGS detected
chrX:49077912:C:T	c.56-1G>A	2	2	2	0	0	0	0	0			0			no matching CES/WGS data	yes	EX	
VPS13C																		
chr15:61856408:G:A*	R3652*	37	37	37	1	0	4	0	1	0/1 (0)		4	0/4 (0)			no	EX	
chr15:61873386:G:A*	R3480*	1	1	1	0	0	0	0	0			0			no matching CES/WGS data	no	EX	
chr15:61874874:A:G*	c.10414+2T>C	1	1	0	0	0	1	1	0			1		1/1	only WGS detected	no	IN	only CES/WGS detected
chr15:61884130:C:A*	E3161X	1	1	0	0	0	1	1	0			1		1/1	only WGS detected	no	IN	only CES/WGS detected
chr15:61908991:C:T*	c.8978+1G>A	1	1	0	0	0	1	1	0			1		1/1	only WGS detected	no	IN	only CES/WGS detected
chr15:61941819:AGAATAATGTTCCATAG:A*	P1794Lfs*9	1	0	0	0	0	1	1	0			0			only CES detected	no	IN	only CES/WGS detected
chr15:61941872:CCAGAT:C*	D1780Gfs*6	1	1	0	0	0	1	1	0			1		1/1	only WGS detected	no	IN	only CES/WGS detected
chr15:61958608:C:G*	G1389R	1	1	0	0	0	1	1	0			1		1/1	only WGS detected	no	IN	only CES/WGS detected
chr15:61977081:C:T*	c.2408+1G>A	3	3	3	0	0	1	0	0			1	0/1 (0)			no	EX	
chr15:61982458:C:A*	c.2029+1G>T	1	1	0	0	0	1	1	0			1		1/1	only WGS detected	no	IN	only CES/WGS detected
SYNJ1																		

chr21:32657714:A:G*	c.2461+2T>C	1	1	0	0	0	1	1	0			1		1/1	only WGS detected	no	IN	only CES/WGS detected
chr21:32678675:G:A*	R494X	1	0	0	0	0	1	1	0			0			only WGS detected	no	IN	only CES/WGS detected
chr21:32695106:C:T*	R219Q	1	1	0	0	0	1	1	0			1		1/1	only WGS detected	no	IN	only CES/WGS detected
chr21:32699922:C:T*	W132X	1	1	0	0	0	1	1	0			1		1/1	only WGS detected	no	IN	only CES/WGS detected
DNAJC6																		
chr1:65366107:C:T*	R152*	1	0	0	0	0	1	1	0			0		1/1	only WGS detected	no	IN	only CES/WGS detected
chr1:65384292:C:T*	R256*	1	1	0	0	0	1	1	0			1			only WGS detected	no	IN	only CES/WGS detected
FBXO7																		
chr22:32475004:T:A*	M1?	1	1	0	1	1	0	0	1		1/1	0			only CES detected	no	IN	only CES/WGS detected
chr22:32491175:C:T*	R321X	4	4	3	1	1	0	0	1		1/1	0				no	IN	validation with CES/WGS
chr22:32493170:C:T*	R345X	5	5	5	1	1	0	0	1	1/1 (100)		0				no	IN	validation with CES/WGS
chr22:32495510:C:T*	Q388X	1	1	0	0	0	1	1	0			1		1/1	only WGS detected	no	IN	only CES/WGS detected
chr22:32498165:C:CAA*	R403Kfs*11	1	1	0	0	0	1	1	0			1		1/1	only WGS detected	no	IN	only CES/WGS detected
chr22:32498453:C:T*	R498*	2	2	2	0	0	0	0	0			0					EX	
JAM2																		
chr21:25702257:C:T*	R229*	1	1	0	0	0	1	1	0			1		1/1	only WGS detected	no	IN	only CES/WGS detected

* Variants marked with an asterisk reflect variants identified in the heterozygous state.
